# Meta-analysis identifying gut microbial biomarkers of Qinghai-Tibet Plateau populations and the functionality of microbiota-derived butyrate in high-altitude adaptation

**DOI:** 10.1080/19490976.2024.2350151

**Published:** 2024-05-07

**Authors:** Hongwen Zhao, Longjie Sun, Jiali Liu, Bin Shi, Yaopeng Zhang, Ci-Ren Qu-Zong, Tsechoe Dorji, Tieyu Wang, Hongli Yuan, Jinshui Yang

**Affiliations:** a State Key Laboratory of Animal Biotech Breeding, College of Biological Sciences, China Agricultural University, Beijing, China; b Key Laboratory of Environmental Nanotechnology and Health Effects Research, Research Center for Eco-Environmental Sciences, Chinese Academy of Sciences, Beijing, China; c State Key Laboratory of Tibetan Plateau Earth System, Resources and Environment (TPESRE), Institute of Tibetan Plateau Research, Chinese Academy of Sciences, Beijing, China; d College of Ecology and Environment, Tibet University, Tibet, China; e Guangdong Provincial Key Laboratory of Marine Disaster Prediction and Prevention, Shantou University, Shantou, China

**Keywords:** Qinghai-Tibet plateau, gut microbiota biomarker, butyrate, high altitude adaptation, HIF-1α

## Abstract

The extreme environmental conditions of a plateau seriously threaten human health. The relationship between gut microbiota and human health at high altitudes has been extensively investigated. However, no universal gut microbiota biomarkers have been identified in the plateau population, limiting research into gut microbiota and high-altitude adaptation. 668 16s rRNA samples were analyzed using meta-analysis to reduce batch effects and uncover microbiota biomarkers in the plateau population. Furthermore, the robustness of these biomarkers was validated. Mendelian randomization (MR) results indicated that Tibetan gut microbiota may mediate a reduced erythropoietic response. Functional analysis and qPCR revealed that butyrate may be a functional metabolite in high-altitude adaptation. A high-altitude rat model showed that butyrate reduced intestinal damage caused by high altitudes. According to cell experiments, butyrate may downregulate hypoxia-inducible factor-1α (HIF-1α) expression and blunt cellular responses to hypoxic stress. Our research found universally applicable biomarkers and investigated their potential roles in promoting human health at high altitudes.

## Introduction

Over 140 million people reside in permanently elevated areas 2500 meters above sea level.^1^ The population of the Qinghai-Tibet Plateau exceeds 9 million,^2^ while the area draws over 42 million tourists annually.^3^ However, exposure to high altitudes can give rise to various illnesses,^4^ the most common of which are gastrointestinal symptoms caused by intestinal damage.^5^ Additionally, damage to the intestinal tract can result in bacterial translocation.^6^ Gut microbiota translocation may injure other organs^7^ and produce systemic inflammation.^8^ Nevertheless, Tibetans have successfully adapted to harsh high-altitude environments.^9^ Understanding the mechanisms by which high-altitude populations adapt to hostile environments is thus crucial for the prevention and treatment of high-altitude disorders.

After long-term adaptive evolution, Tibetans have developed a unique resilience to mountain sickness via *Endothelial PAS domain protein* 1 (*EPAS*1) and *Egl-*9 *family hypoxia-inducible factor* 1 (*EGLN*1)-mediated attenuated erythropoietic response.^1,10,11^ However, the specific mechanisms by which Tibetans adapt to high-altitude environments have yet to be understood. Gut microbiota may also contribute to the host’s adaptation to harsh plateau environments.^12^ Germ-free mice transplanted with Tibetan pig fecal samples have better intestinal development than mice transplanted with Yorkshire or Rongchang pig fecal samples.^13^ In another study, recipient mice developed severe intestinal damage after receiving gut microbiota transplants from donor mice living at a high altitude.^14^ Previous research has indicated that altitude-associated alterations in gut microbiota can directly affect host health. Nevertheless, little is known about the role of gut microbiota in the health of high-altitude populations, and it is unclear whether there is a causal relationship between gut microbiota and the phenomenon of adaptation to high altitudes.

The identification of critical bacteria is a core issue in gut microbiota research. Guo et al.^15^ revealed the mechanism by which the gut microbiota assists in host radiation resistance by identifying the critical gut microbiota of Lachnospiraceae. Wang et al.^16^ found a mechanism for treating obesity and metabolic disorders by identifying the key bacterial strain, *Parabacteroides distasonis*. However, due to confounding factors such as geographical spread, ethnic differences, lifestyle, and sample sequencing, obtaining the key bacteria of the plateau population remains a significant difficulty in revealing how the gut microbiota assists in high-altitude adaptation. Identifying disease-related gut microbiota biomarkers by integrating and analyzing public databases has become widely utilized in medicine, particularly for colorectal cancer (CRC) gut microbiota biomarkers.^17,18^ This approach reduces the influence of confounders across studies and is critical for early disease diagnosis, targeted treatment, and mechanistic investigation. Therefore, integrating gut microbiota data from high-altitude populations and identifying gut microbiota biomarkers specific to these populations will help us understand the mechanisms underlying high-altitude adaptation from the perspective of the gut microbiota.

This study comprehensively analyzed 688 publicly available gut microbiome datasets from 18 Chinese provinces. After correcting for batch effects and ethnic heterogeneity, gut microbiome biomarkers were identified in populations living in the plateau region. Study-to-study transfer validation and leave-one-dataset-out (LODO) were used to prove the robustness of the biomarkers. Furthermore, causal relationships between the gut microbiota and indicators related to polycythemia, such as erythrocyte count, hemoglobin concentration, and hematocrit, were observed using MR. We also found that butyrate, a metabolite possibly derived from Ruminococcaceae and Lachnospiraceae biomarkers,^19^ might reduce Sprague-Dawley (SD) rat intestinal injury caused by high-altitude environments and NCM460 cell damage in simulated hypoxic conditions. Moreover, this study found parallels in the energy metabolic-related gene-blunted phenomenon between hypoxic NCM460 cells treated with butyrate and hypoxic cells of adapted Tibetan human umbilical vein endothelial cells (HUVECs) obtained from the GEO database.^20^ Therefore, this discovery may address the issue of human adaptation to high altitudes from the perspective of the gut microbiota, promoting the health of the high-altitude population.

## Results

### The identification and verification of gut microbiota biomarkers in plateau population based on 16s rRNA data

#### Sample characteristics

**Figure 1.**
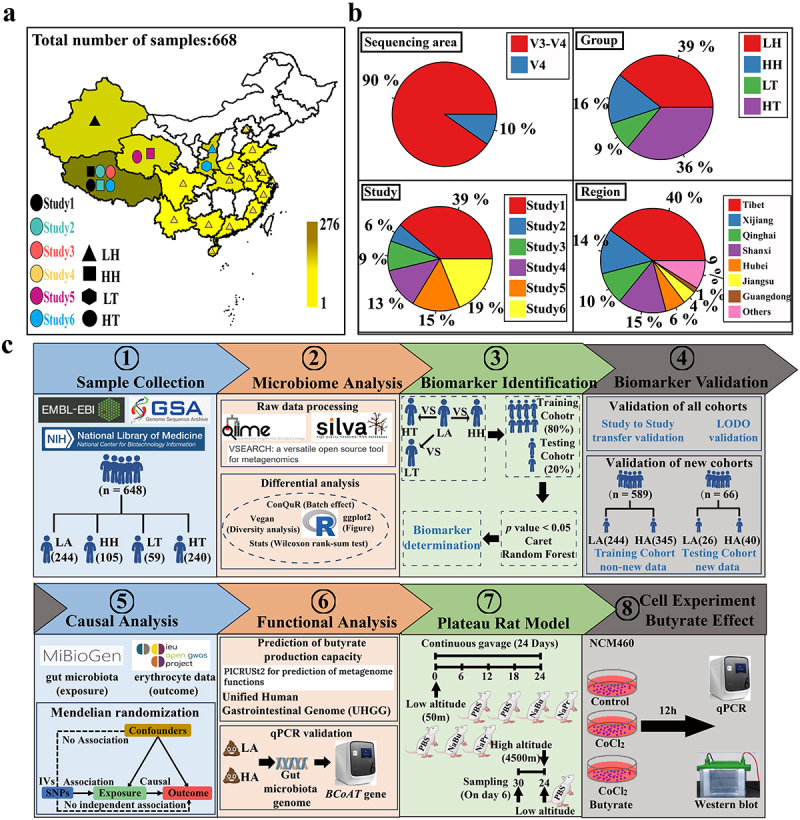
Sample integration and analysis process.

Six hundred and sixty-eight raw datasets were obtained from 18 provinces in China (Figure 1(a,b)), providing information on the subjects’ essential characteristics, disease, diet, and lifestyle (Methods section of the six investigations). This included 263 samples from low-altitude populations (LH), 105 Han samples from high-altitude populations (HH), 60 Tibetan samples from low-altitude populations (LT), and 240 Tibetan samples from high-altitude populations (HT) (Supplementary Table S1). While the ethnicity of some samples (33.84%) is unclear, these samples are likely to include Han individuals, as 91.11 of the Chinese population is Han.^21^ After excluding low-quality samples, we had 647 samples and 26,020 feature sequences, with an average read count of 48,891 per sample. The full analysis process of the samples is shown in Figure 1(c).

(a) The geographic distribution of samples from six studies and information on the number of samples from each region is shown. (b) The proportion of sample sequencing regions, proportion of groups, proportion of different independent institutes, and proportion of samples in each region, respectively. (c) Workflow: ① Gather 668 16S rRNA samples from databases, categorized into LH, HH, LT, and HT groups. ② Process the raw data using software such as Qiime and vesearch, followed by conducting differential analysis using R packages. ③ Identify gut microbiota biomarkers in high and low-altitude populations using *p*-value, caret, and Random Forest methods. ④ Validate the gut microbiota through LODO, study-to-study transfer validation, and new cohorts. ⑤ Utilize Mendelian randomization to infer causal relationships between gut microbiota and high-altitude adaptation. ⑥ Predict and quantify the butyrate production capacity of gut microbiota in high and low-altitude populations. ⑦ Validate the impact of butyrate using a high-altitude rat model. ⑧ Investigate the mechanism of action of butyrate through cell experiments

#### Batch effect interference and adjustment

To evaluate batch effects among samples from distinct cohorts, we used the methodology illustrated in Supplementary Figure S1. We used Shannon Index, observed operational taxonomic units (OTUs) and evenness indices to evaluate the alpha diversity of the sample. We found that the Shannon index of the high-altitude samples was lower than that of the low-altitude samples (Supplementary Figure S2(a)). However, the blocked Wilcoxon Rank sum test revealed that only the Shannon index differed significantly between LH and HT (Supplementary Figure S2(a)). The observed OTUs were used to characterize sample richness. The observed OTUs and evenness indices matched those obtained from the Shannon index (Supplementary Figure S2(b)). Consequently, these results show the occurrence of significant batch effects among data from various study cohorts.

Subsequently, we filtered the samples according to the criteria outlined in Supplementary Figure S1. However, Shannon indices, observed OTUs, and evenness indices showed that batch effects exaggerated or masked these differences (Supplementary Figure S2(c,d)). Beta diversity using PCoA (Bray-Curtis method) revealed a clear clustering trend by “Study” (Supplementary Figure S2(e)). Evaluating the differences in Axis 1 and Axis 2 of PCoA among different “Group” and “Study” treatments showed that although differences between “Group” were considerable, differences between “Study” were also significant (Supplementary Tables S2 and S3). Such differences could affect the reliability of the subsequent analysis results.

**Figure 2.**
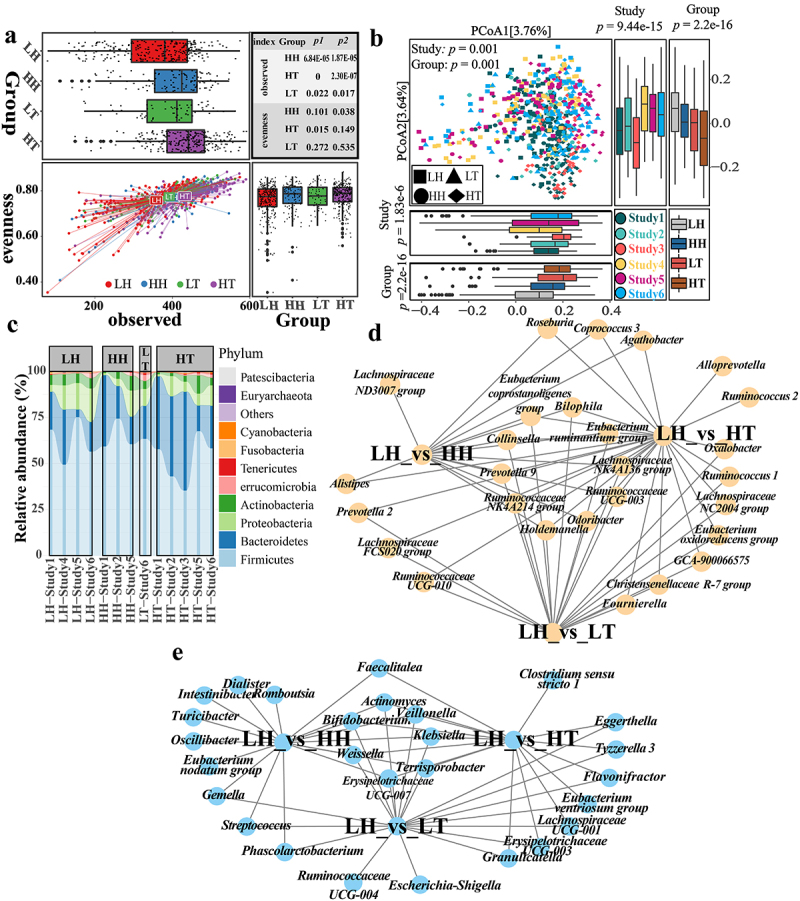
Differences in gut microbiota between high- and low-altitude people.

To mitigate these issues, we processed samples using the ConQuR method^22^ to account for batch effects. After adjusting for batch effects, the Shannon index showed consistent significant both before and after the blocked Wilcoxon Rank sum test (Supplementary Figure S2(f)). These results would suggest that the Shannon index of the gut microbiota in the high-altitude Han and Tibetan populations was significantly higher than that in the low-altitude Han population. There was no significant difference between LH and LT (Supplementary Figure S2(f)). The difference between LH, HH, and HT was mainly due to richness, as the high-altitude population had a significantly higher OTU gut microbiota richness than the low-altitude population. In contrast, differences in the evenness index were minor (Figure 2(a)). These results suggest a unique microbiota in the high-altitude population. Moreover, although the PERMANOVA results showed significant differences between “Study” (Figure 2(b)), comparing the differences between Axis 1 and Axis 2 of PCoA showed that certain spurious differences between different “Study” treatments disappeared or decreased (Supplementary Table S4). In contrast, variations between diverse “Group”s persisted (Supplementary Table S5). Additionally, the trend of sample clustering by “Study” disappeared following beta diversity analysis (Figure 2(b)). In conclusion, beta diversity results showed significant differences in gut microbiota composition between high- and low-altitude populations. Hence, using either the ConQuR or the blocked Wilcoxon Rank sum test, we preserved intergroup differences while mitigating batch effects across diverse “Study” treatments. Finally, to avoid excessive data manipulation that could arise from using both techniques concurrently, we decided to use the ConQuR method alone for subsequent analysis.

(a) Richness index and evenness index scatter plot after filtering some OTUs and ConQuR processing (LH, *n* = 243; HH, *n* = 105; LT, *n* = 59; HT, *n* = 240). The box plot on the top indicates the observed index results of different groups, while the box plot on the right indicates the evenness index results of samples of other groups. The table on the top right shows the difference between groups in observed and evenness index compared with LH. *p* 1 was from the Wilcoxon Rank sum test. *p* 2 was from the blocked Wilcoxon Rank sum test. (b) PCoA analysis of samples after filtering some OTUs and ConQuR processing (LH, *n* = 243; HH, *n* = 105; LT, *n* = 59; HT, *n* = 240). Beta diversity was based on Bray-Curtis Dissimilarity. *p* value of the PCoA was from PERMANOVA (999 permutations). The *p* value of the boxplot was from the Kruskal test. (c) The relative abundance and variation trend of phyla-level species in 6 study cohorts. Draw the picture using unprocessed data. The image is drawn through the microbiome database (http://egcloud.cib.cn/). (d, e) The relative abundance in genera level of HH, HT, and LT were significantly increased (d) or decreased (e) compared to LH. FDR-corrected *p* value was from the Wilcoxon Rank sum test.

We also assessed the impact of age and sex on the gut microbiota using data from Studies 3 and 4. There was no significant correlation between age and the Shannon index, evenness index, or observed OTUs of the gut microbiota in Studies 3 (HT) and 4 (LH) (Supplementary Figure S3(a–f)). Similarly, no notable differences in these parameters were observed between the sexes in Studies 3 and 4 (Supplementary Figure S4(a–f)). This indicates that in our study, the age and sex of both Han and Tibetan samples may have a minor impact on the gut microbiota.

### Characteristics of gut microbiota composition in a high-altitude population

Firmicutes, Bacteroidetes, Proteobacteria, and Actinobacteria were the dominant phyla in the gut microbiota (Figure 2(c)). Tenericutes, Euryarchaeota, and Patescibacteria were the only phyla that differed significantly between LH and LT. However, gut microbiota changes in high-altitude populations followed a more consistent trend, with a significant enrichment of Bacteroidetes and a significantly lower proportion of Proteobacteria, Actinobacteria, Fusobacteria, and other phyla (Supplementary Figure S5(a)). However, there were inconsistent trends in the relative abundances of the most abundant Firmicutes between HH and HT. Compared with LH, HH displayed a significantly higher relative abundance of Firmicutes, whereas HT exhibited a significantly lower relative abundance. This could assist Tibetans residing at high altitudes to adapt more effectively to challenging plateau environments.

At the genus level, we identified 42, 74, and 59 significantly different genera between LH_vs_HH, LH_vs_HT, and LH_vs_LT. As depicted in Supplementary Figure S5(b), eliminating altitudinal differences did not reduce the differences in gut microbiota composition between Tibetan and low-altitude Han populations. Only ten unique genera were identified between LH and HH, suggesting that low-altitude Han populations’ gut microbiota changes when entering high-altitude regions. Specifically, the gut microbiota of Han individuals may change to resemble that of the plateau Tibetan. Additionally, twenty-one genera exhibited similar trends in both LH_vs_HH and LH_vs_HT, with 16 being consistent with LH_vs_LT. Similarly, a study found that Han individuals returning from high-altitude to low-altitude regions for three months had gut microbiota compositions more akin to the high-altitude population rather than reverting to the low-altitude Han population.^23^ These findings imply that individuals migrating from high- to low-altitude regions may retain specific gut microbiota characteristics found in high-altitude populations. Fourteen genera were significantly higher in high-altitude populations (HH and HT), including Ruminococcaceae NK4A214, *Prevotella* 9, Lachnospiraceae NK4A136, and Ruminococcaceae UCG-003 (Figure 2(d)). Seven genera, including *Bifidobacterium*, *Veillonella*, and *Klebsiella*, were significantly more abundant in the low-altitude Han population (Figure 2(e)). *Roseburia* and *Coprococcus* 3 from the Lachnospiraceae, and *Ruminococcus* 2 and *Butyricimonas* from the Ruminococcaceae were significantly enriched in the high-altitude Han and Tibetan populations, respectively. Lachnospiraceae and Ruminococcaceae are the primary short-chain fatty acid (SCFA)-producing probiotic families.^19^ The abundance of Lachnospiraceae was higher in the HH group than in the LH group (FDR-corrected *p* = 0.056). Ruminococcaceae were also significantly more prevalent in the Tibetan population (Supplementary Figure S5(c)).

### High-altitude human gut microbiota biomarker identification

**Figure 3.**
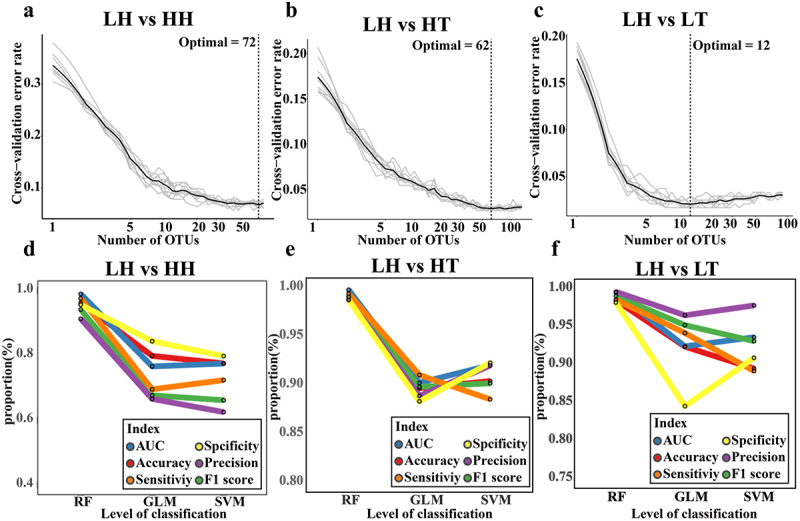
Identification of gut microbiota biomarkers in plateau population.

We used the methodology illustrated in Supplementary Figure S1 to identify and validate gut microbiota biomarkers in plateau populations. The results showed that model error was minimized at 72, 62, and 12 biomarkers for LH vs. HH, LH vs. HT, and LH vs. LT, respectively (Figure 3(a–c)). These OTUs were considered gut microbiota biomarkers for HH, HT, and LT against LH (Supplementary Figs. S6–8). Regarding the gut microbiota biomarkers of the high-altitude population, these OTUs mainly belonged to Ruminococcaceae and Lachnospiraceae (Supplementary Figure S6(a) and S7(a)). The bacterial genera *Dorea*, *Roseburia*, *Prevotella* 9, and *Faecalibacterium* were relatively abundant in the high-altitude population. (Supplementary Figure S6(b,c) and S7(b,c)). Although the bacterium *Faecalibacterium* contains several OTUs, some of which are LH-enriched, *Faecalibacterium* is more abundant in high-altitude populations. To verify the ability of these OTUs to identify gut microbiota biomarkers, we created three classification models using Random Forest (RF), Generalized Linear Model (GLM), and Support Vector Machine (SVM). The results indicated that in LH vs. HH, LH vs. HT, and LH vs. LT, the models built using gut microbiota biomarkers performed well, with the RF model performing the best (Figure 3(d–f) and S9(a – c), and Supplementary Data 1–6), suggesting that RF may be more suitable for constructing the classifier. The average area under the curve (AUC) of the RF model for distinguishing HH from LH was 0.990 ± 0.010 (accuracy: 0.962 ± 0.028, sensitivity: 0.976 ± 0.038, specificity: 0.956 ± 0.038, precision: 0.913 ± 0.074, F1 score: 0.942 ± 0.043, Supplementary Data 1). The average AUC of the RF model to distinguish HT from LH was 0.998 ± 0.002 (accuracy: 0.991 ± 0.007, sensitivity: 0.994 ± 0.010, specificity: 0.988 ± 0.014, precision: 0.988 ± 0.013, F1 score: 0.991 ± 0.007, Supplementary Data 2). The average AUC of the RF model to distinguish LT from LH was 0.995 ± 0.007 (accuracy: 0.985 ± 0.023, sensitivity: 0.985 ± 0.030, specificity: 0.982 ± 0.036, precision: 0.996 ± 0.08, F1 score: 0.990 ± 0.015, Supplementary Data 3). Therefore, these gut microbiota biomarkers represent both high- and low-altitude populations.

(a – c) The optimal number of gut microbiota biomarkers for LH_vs_HH (a), LH_vs_HT (b), and LH_vs_LT (c) were determined by random forest. (d – f) The performance of RF, GLM, and SVM classification models constructed by LH_vs_HH (d), LH_vs_HT (e), and LH_vs_LT (f) using the gut microbiota biomarkers, respectively. Blue, red, orange, yellow, purple, and green represent AUC, Accuracy, Sensitivity, Specificity, Precision, and F1 Score, respectively.

### High-altitude human gut microbiota biomarker validation

**Figure 4.**
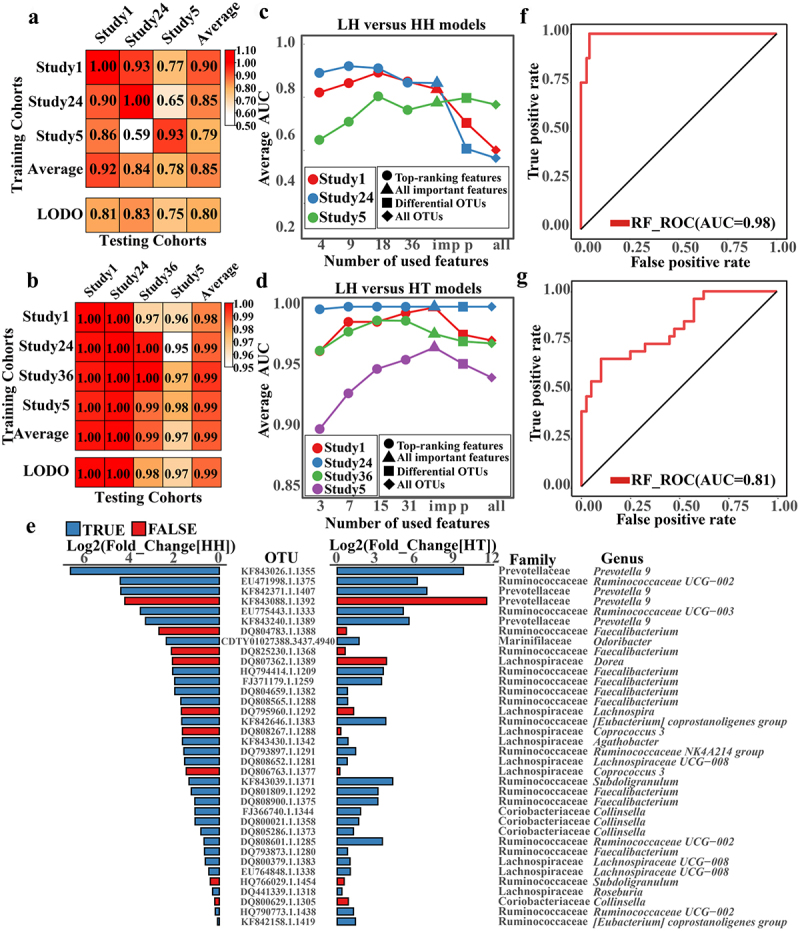
Validation of predictive performance of gut microbiota biomarkers in plateau population.

Among the seven classification levels considered, the model built using OTUs performed more reliably for six indicators (Supplementary Figure S10(a–c) and Supplementary Data 1–3). Subsequently, we assessed the universality and generalizability of the identified gut microbiota across studies using study-to-study and LODO methods. During study-to-study validation, the LH_vs._HH and LH_vs._HT models achieved average AUC values of 0.85 (range: 0.59 to 1) and 0.99 (range: 0.95 to 1), respectively. Similarly, in LODO validation, the LH_vs._HH and LH_vs._HT models attained average AUC values of 0.80 (range: 0.75 to 0.83) and 0.99 (range: 0.97 to 1), respectively (Figure 4(a,b)). These results suggest that gut microbiota biomarkers can be used to differentiate populations at different altitudes. Moreover, the Tibetan gut microbiota has a superior ability to distinguish between populations compared to the plateau Han. This implies more pronounced disparities in the gut microbiota composition between plateau Tibetans and low-altitude Han individuals.

(a,b) Cross-validation matrices of LH_vs_HH (a) and LH_vs_HT (b), respectively. The model was constructed using the RF method with 10-fold cross-validation. The value represents the AUC, and the color depth was positively correlated with the AUC. The top squares represent the result of study-to-study transfer validation, and the bottom squares represent the result of LODO validation. The training and test samples are marked on the left and top, respectively. All samples except for test samples were used as training samples in LODO validation. (c,d) AUC value of LH vs HH (c) and LH vs HT (d) LODO validation classifiers with different characteristics. Circles, triangles, squares and diamonds represent top-ranking biomarkers, all biomarkers, all significantly different OTUs and all OTUs, respectively. The color of the line represents the test set sample. The Wilcoxon Rank sum test calculated the significant difference in OTUs (FDR-corrected *p* value < 0.05). (e) The gut microbiota co-biomarkers of high-altitude population. Fold change is equal to the ratio of the average relative abundance of OTU in HH or HT to the average relative abundance of OTU corresponding to LH. The length of the bar graph is log2 (fold change). The blue bar indicates a significant difference between LH and LT (FDR-corrected *p* value < 0.05), and the red indicates a non-significant difference between LH and LT. (f) ROC curves of LH_vs_HA(High Altitude[HH and HT]). 20% of the total samples were randomly selected as the test set, and the remaining samples were used as the training set, repeated ten times. Only the optimal ROC curve and average AUC values are shown here. (g) A set of newly collected samples (LH, *n* = 26; HA, *n* = 40) was used as a test set to verify the model constructed with LH and HA samples as the training set.

LODO was used to verify the trained model’s specific ability for the plateau population using all OTU, OTU with significant differences, all biomarkers, and some of the top biomarkers as features (Figure 4(c,d) and Supplementary Data 7 and 8). When all OTUs or OTUs with significant differences were used as training model attributes, performance was poor in comparison to the training model that identified biomarkers or top biomarkers. These results support the rationality for the biomarkers identified in this study.

Although there were more pronounced disparities in gut microbiota between LH and HT than between LH and HH, alterations shared by HH and HT may be more relevant for high-altitude adaptation in plateau populations. Consequently, we identified gut microbiota co-biomarkers for LH vs. HH and LH vs. HT. We identified 36 OTUs that were significantly enriched in high-altitude populations (Figure 4(e)). These 36 OTUs were considered gut microbiota co-biomarkers in the plateau population. Notably, of the 36 co-biomarkers, 9 and 18 belonged to Lachnospiraceae and Ruminococcaceae, respectively. When compared to LH, 27 of these co-biomarkers were significantly enriched in LT. The bacteria were identified as *Roseburia*, *Prevotella* 9, Lachnospiraceae UCG-008, Ruminococcaceae UCG-002, Ruminococcaceae UCG-003, Ruminococcaceae NK4A214, *Faecalibacterium*, *Collinsella*, and others. They may be linked to both altitude and ethnicity. Moreover, OTUs identified as *Dorea*, *Lachnospira*, and *Coprococcus* 3 showed no significant differences between the LH and LT groups, indicating that the differences in gut microbiota may be mostly due to altitude.

These 36 gut microbiota co-biomarkers were used to build an RF model that differentiates between high and low-altitude populations, with an impressive AUC value of 0.97 (Figure 4(f) and Supplementary Data 9). Moreover, using the RF model constructed with these 36 co-biomarkers to distinguish between distinct cohort data sets, the AUC value can approach 0.81. (Figure 4(g)). These results indicate that the identified co-biomarkers may successfully distinguish between high-altitude populations and are robust.

Although we identified gut microbiota co-biomarkers in plateau populations, it is uncertain whether there is a causal relationship between gut microbiota, particularly these co-biomarkers, and the adaptation of plateau populations to high altitudes.

### The Mendel randomization analysis based on genome-wide association analysis (GWAS) data

#### Gut microbiota associated with phenotypes is linked to polycythemia

**Figure 5.**
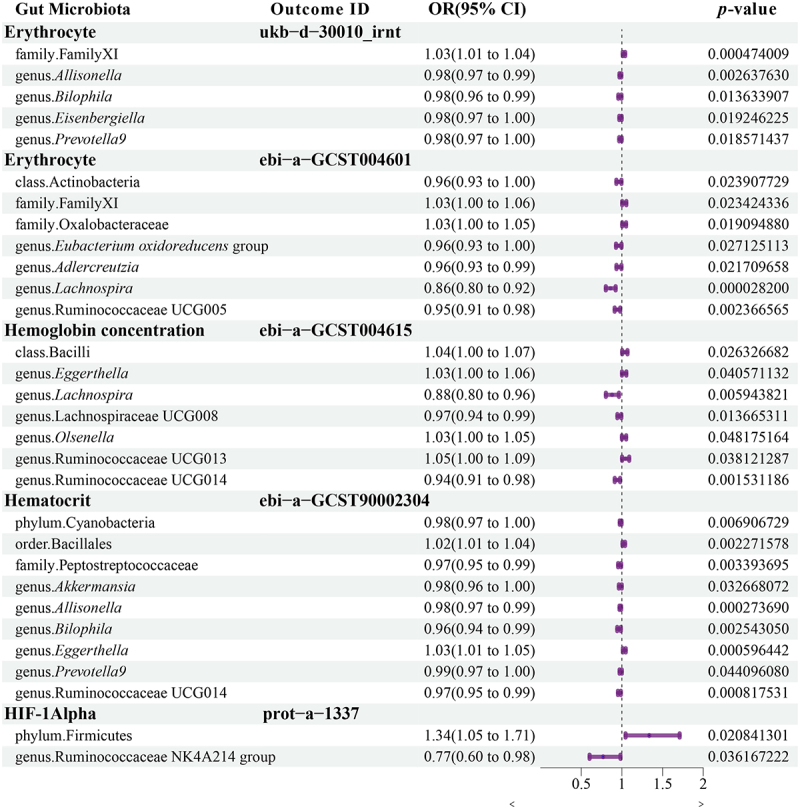
The causal relationship between gut microbiome and altitude adaptation.

Patients with high-altitude polycythemia have significantly higher erythrocyte, hemoglobin, and hematocrit levels.^24^ However, the Tibetan population living at high altitudes is resistant to this increase, lowering the prevalence of polycythemia.^1,9^ To investigate the role of gut microbiota in high-altitude polycythemia, a Mendelian randomization study was conducted to explore the causal relationship between gut microbiota and erythrocyte count, hemoglobin concentration, and hematocrit. As shown in Figure 5, erythrocyte counts were negatively correlated with *Bilophila*, *Prevotella* 9, *Eubacterium oxidoreducens* group, *Lachnospira*, and Ruminococcaceae UCG005. Unlike LH, *Bilophila* and *Prevotella* 9 had significantly higher relative abundances in both HH and HT, with four co-biomarkers within *Prevotella* 9. The *Eubacterium oxidoreducens* group was more abundant in the HT and LT populations, whereas *Lachnospira* had a higher relative abundance in HH, with one co-biomarker within *Lachnospira*. Although Ruminococcaceae UCG005 did not differ significantly between high- and low-altitude populations, two biomarkers were found in Ruminococcaceae UCG005 in LH vs. HT. These findings connect specific gut microbiota biomarkers within high-altitude populations to erythrocyte counts. The composition of the gut microbiota in high-altitude populations contributes to reduced erythrocyte proliferation.

Mendelian randomization results of causal effects between the gut microbiome and polycythemia-associated phenotypes

*Eggerthella* exhibited a positive causal relationship with hemoglobin concentration and was more abundant in the LH than in the Tibetan population (LT and HT). Similarly, Ruminococcaceae UCG013 demonstrated a positive causal relationship with hemoglobin concentration. Although there was no significant difference in Ruminococcaceae UCG013 between the Tibetan and LH populations, a specific OTU within Ruminococcaceae UCG013 was identified as a biomarker that differentiated between LH and HT, with a significantly lower abundance in the HT group. *Lachnospira* and Lachnospiraceae UCG008 had a negative causal relationship with hemoglobin concentration. Notably, a co-biomarker was found to be associated with Lachnospiraceae UCG008 in the high-altitude population. Additionally, seven OTUs from Lachnospiraceae UCG008 were identified as biomarkers for the LH vs. HH comparison, with four displaying significantly higher abundance in HH than in LH.

We discovered that *Bilophila* and *Prevotella* 9 had a negative causal relationship with hematocrit. Similarly, the probiotic *Akkermansia* had a negative causal relationship with hematocrit (HCT). Ruminococcaceae UCG014 demonstrated a negative causal relationship with both hemoglobin concentration and HCT. No differences were observed between *Akkermansia* and Ruminococcaceae UCG014. *Eggerthella* demonstrated a positive causal relationship with both hemoglobin concentration and HCT.

Firmicutes had a positive causal relationship with HIF-1α, In contrast, Ruminococcaceae NK4A214 exhibited a negative causal relationship. Firmicutes was significantly less abundant in HT than in LH, although the Ruminococcaceae NK4A214 group was more abundant in both HH and HT. Furthermore, a species within Ruminococcaceae NK4A214 acts as a co-biomarker. This finding supports the potential role of the gut microbiota in regulating HIF-1α levels among individuals living at high altitudes.

A causal relationship between the gut microbiota and phenotypes associated with polycythemia identified through MR analysis suggests a potential connection between the gut microbiota and high-altitude adaptation.

### Investigating the role of butyrate in high-altitude populations through rat and cellular experiments

#### Enrichment of butyrate-producing gut microbiota in high-altitude populations

**Figure 6.**
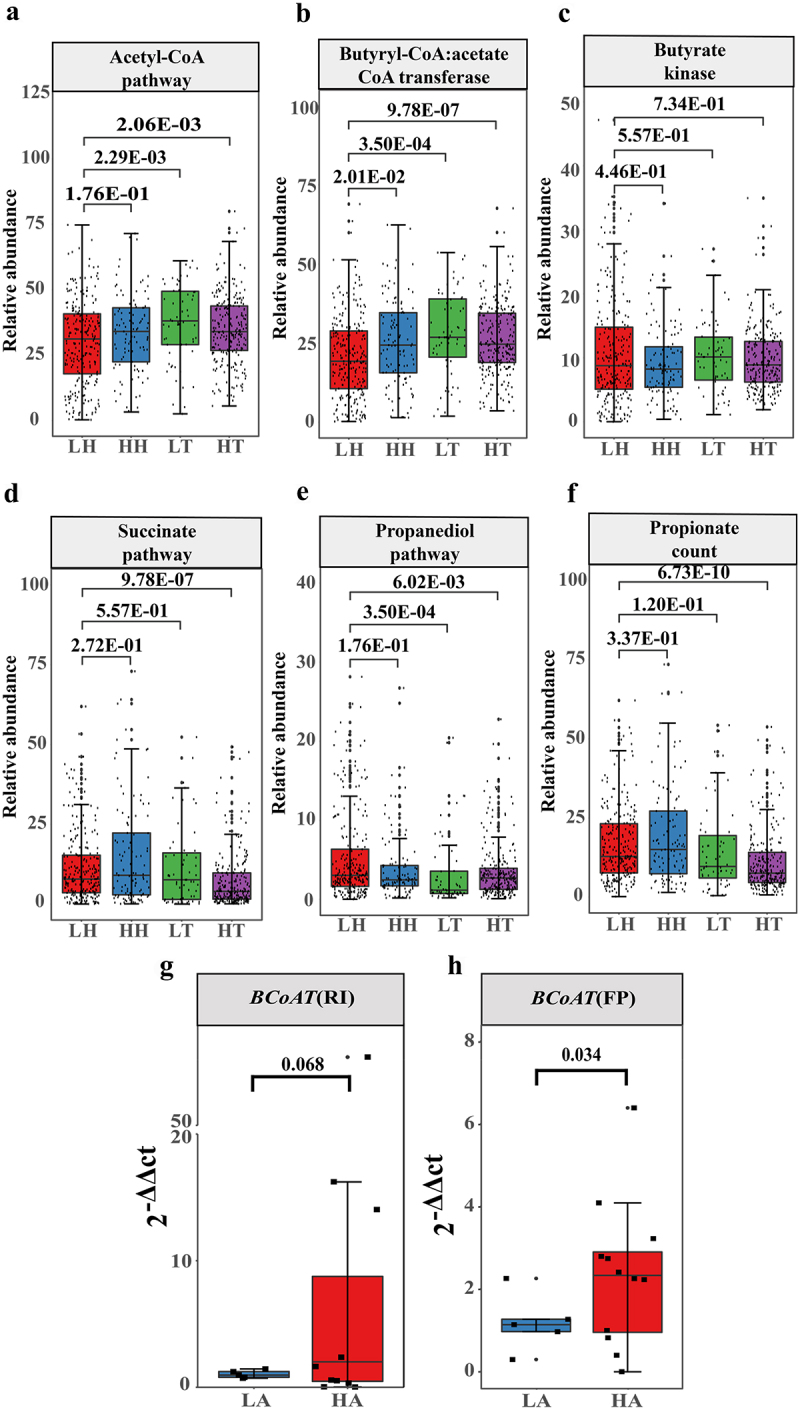
Changes in the abundance of gut microbiome SCFA-producing bacteria in high- and low-altitude population.

The capacity of gut microbiota to produce SCFAs in populations residing at both high and low altitudes was investigated. The gut microbiota primarily synthesizes butyrate via the acetyl-CoA pathway, with butyrate kinase (Buk) and butyryl-CoA:acetate CoA-transferase (BCoTA) being two pivotal genes.^25^ The results showed that compared with LH, Tibetan populations (HT and LT) had significantly higher gut microbiota via the acetyl-CoA pathway (Figure 6(a)). The gut microbiota BCoAT was more abundant in the high-altitude populations (HH and HT) than in the LH population (Figure 6(b)). There was no significant difference in the gut bacterial abundance of HH, HT, and LT Buk compared with LH (Figure 6(c)). Moreover, Relative abundances of propionate-producing bacteria was lower in Tibetan populations (HT and LT) (Figure 6(d–f)). The qPCR results showed a significantly higher abundance of BCoAT in the gut microbiota of the high-altitude population than in the low-altitude population (Figure 6(g,h)). These findings suggest that the gut microbiota of high-altitude populations contain a greater number of butyrate-producing bacteria.

(a) Prediction of the abundance of butyrate-producing bacteria by acetyl-CoA pathway. (b) Prediction of the bacterial abundance of butyryl-CoA: acetate CoA transferase. (c) Prediction of the bacterial abundance of Butyrate kinase. (d) Prediction of the abundance of propionate-producing bacteria by succinate pathway. (e) Prediction of the abundance of propionate-producing bacteria by propanediol pathway. (f) Prediction of the abundance of total propionate-producing bacteria. (c) – (f) FDR-corrected *p* value was from the Wilcoxon Rank sum test. (g,h) qPCR results. The difference between the abundance of BCoAT in LH (*n* = 5) and HA (*n* = 12) is shown in (g) BCoAT primers designed according to R. *intestinalis*. (h) BCoAT primers designed according to *F.prausnizii*. The 2^−ΔΔCT^ denotes the abundance. *p* values were obtained from a one-sided t-test.

#### Butyrate relieved intestinal damage caused by a high-altitude environment

**Figure 7.**
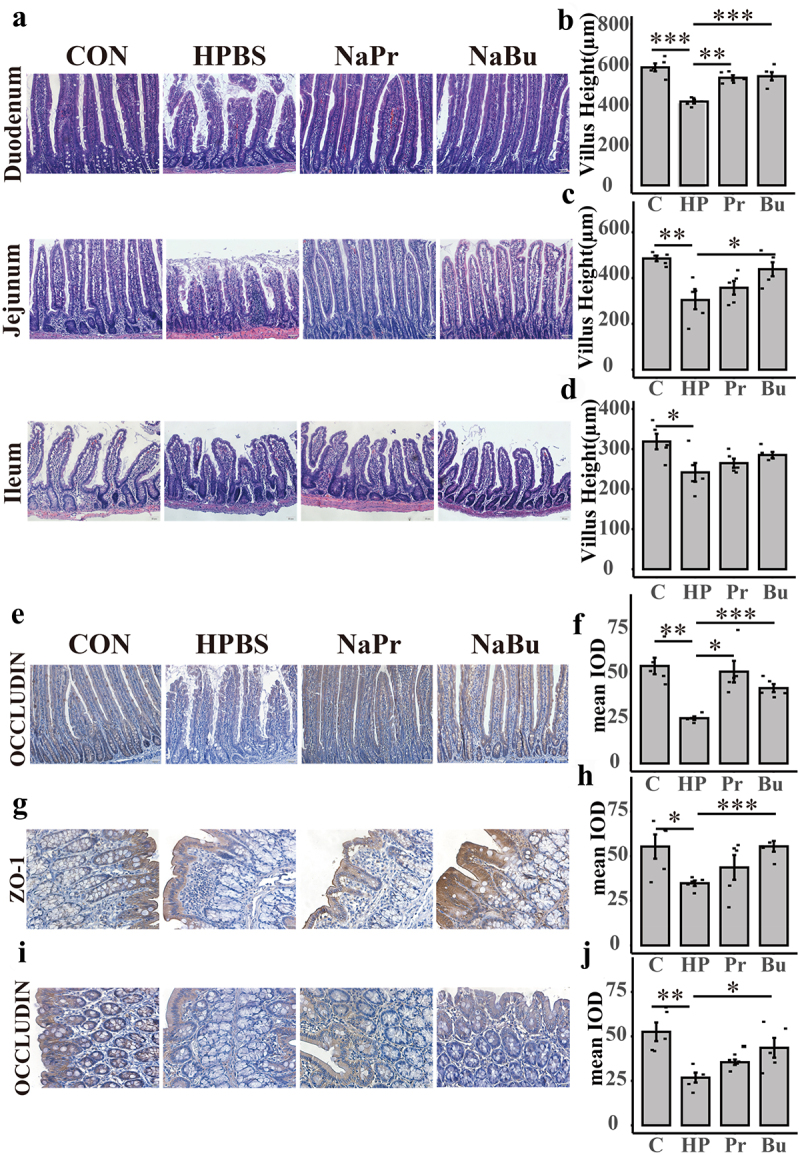
SCFA relieved intestinal damage caused by the plateau environment.

To ascertain the potential protective effects of butyrate in high-altitude environments, we examined its effect on the gastrointestinal tract of SD rats at high altitudes (Figure 1(c)). We also investigated the effects of propionate. Histological analysis revealed notable damage to the small intestine of SD rats in response to the high-altitude environment (Figure 7(a)). Specifically, the high-altitude propionate group (NaPr) and high-altitude butyrate group (NaBu) had a significantly alleviated duodenal villi shortening caused by the high-altitude environment. NaBu also significantly alleviated jejunal villi shortening caused by the high-altitude environment. However, neither NaPr nor NaBu showed any relief in the ileum (Figure 7(a–d)). The villi/crypt in the duodenum, jejunum, and ileum showed similar results (Supplementary Figure 11(a–c)).

(a) HE staining of duodenum, jejunum, and ileum of the CON, HPBS, NaPr, and NaBu groups (*n* = 5, bar = 50 μm). (b–d) The villi length of the duodenum, jejunum, and ileum, respectively. (e) Immunohistochemical staining of occludin in duodenal tissue sections of CON, HPBS, NaPr, and NaBu groups (bar = 50 μm). (f) Mean IOD of occludin in the duodenum. (g,i) Immunohistochemical staining of ZO-1(g) and occludin(i) in colon tissue sections of CON, HPBS, NaPr, and NaBu groups (bar = 20 μm). (h,j) Mean IOD of ZO-1(h), occluding(j) in the colon. Con/C: control group; HPBS/HP: high-altitude PBS group; NaBu/Bu: high-altitude butyrate group; NaPr/Pr: high-altitude propionate group. The results are expressed as Mean±SE (*n* = 5). “*” stands for significant difference. *p < 0.05. **p < 0.01. ***p < 0.001. ****p < 0.0001. The *p* value is calculated by t-test.

Additionally, immunohistochemistry results indicated that NaPr and NaBu could lessen the downregulation of occludin expression in the duodenum caused by the high-altitude environment, thereby preserving intestinal barrier integrity (Figure 7(e,f)). Moreover, the high-altitude environment reduced colonic Zonula occludens 1 (ZO-1) and occludin expression compared to that in rats treated with high-altitude phosphate buffer group(HPBS). Rats treated with butyrate exhibited a significant increase in the expression of ZO-1 and occludin, resulting in the relief of colonic intestinal barrier damage. However, rats treated with propionate did not show any relief from colonic injury (Figure 7(g–j)). Furthermore, we observed that the HPBS group had significantly lower butyrate levels in rat feces than the Con group, whereas the butyrate gavage group had restored levels in the rat intestines (Supplementary Figure S11(s)). In summary, our study confirmed the significant role of butyrate in alleviating intestinal injury caused by high altitudes.

#### Butyrate reduces the expression of HIF-1α

**Figure 8.**
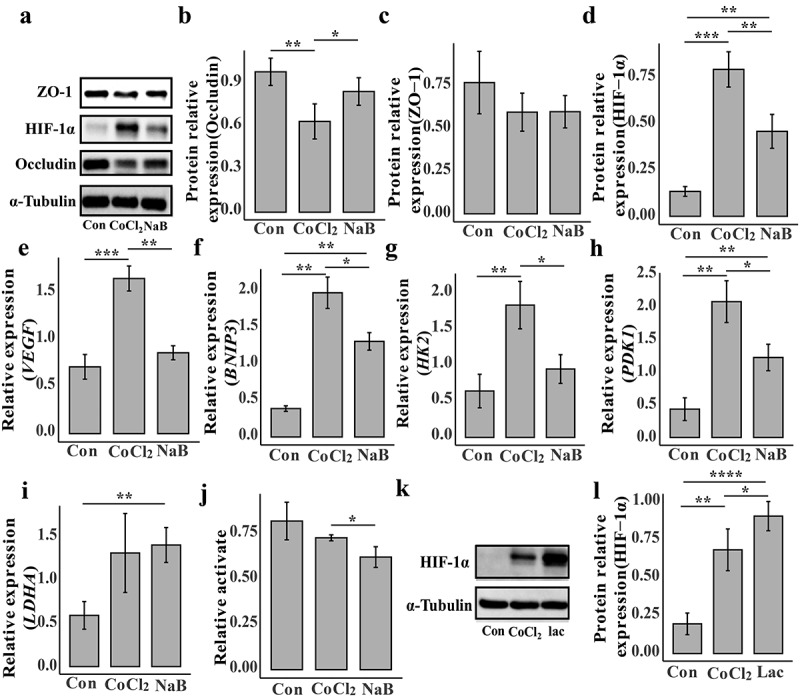
Butyrate alleviates intestinal damage caused by hypoxia by down-regulating HIF-1α in NCM460.

To investigate the mechanism by which butyrate protects against intestinal injury caused by high-altitude hypoxia, we treated NCM460 cells with CoCl_2_ to induce cellular hypoxia and investigated the underlying mechanism. After 24 h, CoCl_2_ caused dose-dependent cellular damage (Supplementary Figure S12(a)), whereas butyrate below 1 mM had no proliferative or inhibitory effects on the cells (Supplementary Figure S12(b)). At the transcriptional level, we found that butyrate administration significantly increased the expression of intestinal barrier *occludin* in hypoxic cells (Supplementary Figure 13(a)). However, *ZO-1* expression did not significantly change (Supplementary Figure 13(b)). Similarly, at the protein level, occludin was significantly downregulated in hypoxic cells; however, after butyrate treatment, occludin expression was recovered (Figure 8(a,b)). There was also no significant difference in the ZO-1 protein levels (Figure 8(c)). Previous studies found that hypoxia causes apoptosis. *Caspase-*3 gene expression decreased significantly after butyrate treatment (Supplementary Figure 13(c)). These results indicated that butyrate plays a beneficial role in cells under hypoxic conditions.

(a) Western blot of ZO1, HIF-1α and occludin. Alpha-tubulin is the internal reference protein. (b) Densitometry quantification of occludin level by Western blot (Con: *n* = 4; CoCl_2_: *n* = 4; NaB: *n* = 4). (c) Densitometry quantification of ZO-1 level by Western blot (Con: *n* = 4; CoCl_2_: *n* = 4; NaB: *n* = 4). (d) Densitometry quantification of HIF-1α level by Western blot (Con: *n* = 4; CoCl_2_: *n* = 4; NaB: *n* = 4). (e) mRNA expression of *VEGF* (Con: *n* = 3; CoCl_2_: *n* = 3; NaB: *n* = 3). (f) mRNA expression of *BNIP*3 (Con: *n* = 3; CoCl_2_: *n* = 3; NaB: *n* = 3). (g) mRNA expression of *HK2* (Con: *n* = 3; CoCl_2_: *n* = 3; NaB: *n* = 3). (h) mRNA expression of *PDK*1 (Con: *n* = 3; CoCl_2_: *n* = 3; NaB: *n* = 3). (i) mRNA expression of *LDHA* (Con: *n* = 3; CoCl_2_: *n* = 3; NaB: *n* = 3). (j) LDHA relative activity (Con: *n* = 4; CoCl_2_: *n* = 4; NaB: *n* = 4). (k) Western blot of HIF-1α. Alpha-tubulin is the internal reference protein. (l) Densitometry quantification of HIF-1α level by Western blot (Con: *n* = 4; CoCl_2_: *n* = 4; lactate: *n* = 4). “*” stands for significant difference. *p < 0.05. **p < 0.01. ***p < 0.001. ****p < 0.0001. The *p* value is calculated by t-test.

Overexpression of HIF-1α/NF-κB/STAT1 in high-altitude environments may be involved in mediating colon tissue damage.^26^ Our study showed a significant increase in HIF-1α expression in hypoxic cells than control, and butyrate could alleviate the increase of HIF-1α (Figure 8(a,d)). We examined *vascular endothelial growth factor (VEGF)* and *BCL*2*/adenovirus E*1*B* 19*kDa interacting protein* 3 *(BNIP3)*, which are target genes of HIF-1, and found that their transcription levels are similar to changes in HIF-1α expression (Figure 8(e,f)). Butyrate can thus regulate HIF-1α in low-oxygen environments, facilitating cellular adaptation to hypoxia.

#### Butyrate downregulates HIF-1α expression by reducing lactate dehydrogenase-A (LDHA) activity, effectively blunting the glycolytic pathway

To determine how acute hypoxia is regulated at the cell gene level, we obtained transcriptome data from the GEO database for Tibetan and Han HUVEC under normal and hypoxic conditions at different times. On the first day of hypoxia, glycolysis and the HIF-1 signaling pathway were upregulated in the Han and Tibetan groups (Supplementary Figure S14(a,b)). However, on the fifth day, glycolysis and HIF-1 signaling pathway upregulation disappeared (Supplementary Figure S14(c,d)). These results suggest that in the early stages of high-altitude hypoxia, the body may mainly respond to hypoxia through HIF-1 while the glycolytic pathway is reshaped. Therefore, by analyzing the changes in gene transcription during glycolysis, the Tricarboxylic Acid Cycle (TCA), and oxidative phosphorylation pathways of Tibetan and Han cells at different times, we found that the trends of these changes were almost identical. On the first day, the glycolysis pathway was significantly upregulated, but there was essentially no difference in TCA cycle genes (Supplementary Figure S15). Most oxidative phosphorylation-related genes were significantly down-regulated, indicating that low-oxygen conditions may reduce aerobic respiration while increasing anaerobic respiration. We found a significant upregulation of the *pyruvate dehydrogenase kinase* 1 *(PDK*1) gene (PDK1 inhibits pyruvate dehydrogenase complex activity, thereby impeding the conversion of pyruvate to acetyl-CoA), which may limit pyruvate entry into the TCA cycle and promoting lactate production. Interestinglyly, under hypoxic conditions, most gene changes in the glycolysis and oxidative phosphorylation pathways in Tibetan cells were smaller than in Han cells. This may be related to the habituation of hypoxic responses in the Tibetan population. HIF-1 is thought to regulate the key genes of glucose anaerobic glycolysis *hexokinase* 2 *(HK*2), lactate production *lactate dehydrogenase A* *(LDHA)*, and *PDK*1. We found that the change in the expression of these three genes in Tibetan cells was significantly lower than that in Han cells during the same low-oxygen treatment period (Supplementary Figure S16(a–c)). Therefore, we examined the effect of butyrate on the transcription levels of these genes and found that, compared to the control group, *HK*2, *LDHA*, and *PDK*1 were upregulated after hypoxic treatment (Figure 8(g–i)). Butyrate treatment reduced *HK*2 and *PDK*1 transcription (Figure 8(g,h)); however, butyrate did not affect LDHA transcription (Figure 8(i)). Moreover, we observed intracellular lactate accumulation after hypoxia treatment, but this was significantly reduced after butyrate treatment (Supplementary Figure S16(d)). We also found thatbutyrate treatment significantly downregulated of LDHA activity in hypoxic cellit (Figure 8(j)). These results indicated butyrate may promote the blunting of cells to hypoxia by reducing HIF-1α in low-oxygen conditions, avoiding excessive stress responses of cells to hypoxia, and helping cells adapt to low-oxygen environments. Previous reports suggest that lactate can directly bind to prolyl hydroxylase-2 (PHD2) and increase HIF-1α stability.^27^ Therefore, it is hypothesized that the upregulation of the glycolytic pathway leads to lactate accumulation, resulting in the increased expression of HIF-1α in hypoxic cells. Additionally, our experiments demonstrate that adding exogenous lactate further enhances the stability of HIF-1α in hypoxic cells (Figure 8(k,l)). In conclusion, it is speculated that butyrate can prevent excessive accumulation of HIF-1α by inhibiting LDHA activity and reducing lactate accumulation.

## Discussion

### Batch effect in microbiome studies

With the maturation of sequencing technologies, the pitfalls of microbiome research have gradually been exposed. Neglecting confounders can significantly compromise the credibility of results.^28^ Age and sex are confounding factors that should be considered. We evaluated the effect of age and sex on the gut microbiota using data from Study 3 and 4, and no significant effects were found. Similarly, another study found no significant correlation between the alpha diversity of the gut microbiota and age in the over three age group across various ethnic groups of Chinese residents.^29^ Thus, age and sex may exert only a minor influence on the gut microbiota. Furthermore, batch effects may be the most pronounced confounders. Two batches of experimental rats with identical genetic backgrounds were obtained from the same supplier and raised in the same environment. There are considerable differences in the gut microbiota resulting from batch effects.^30^ Although meta-analysis can help mitigate the impact of biological and technical confounders, identifying early colorectal cancer (CRC) gut microbiota biomarkers through meta-analysis reveals that the variance of ASV explained by “study” was more significant than that by disease status and other potential confounders such as age and sex.^18^ Therefore, we used ConQuR^22^ to correct for batch effects in the microbiome data. The updated results show a significant reduction in the impact of batch effects while preserving the differences between the various groups. This indicates that when studying the gut microbiota in plateau populations, multiple confounders must be considered, particularly the influence of batch effects. Although we controlled for batch effects, the lack of data on the age, sex, and dietary habits of all samples hindered our ability to thoroughly examine the correlation between the gut microbiota and age, sex, and diet. Therefore, these aspects should be considered in future microbiome studies.

High-altitude Tibetan gut biomarkers had better discrimination ability than the model trained using high-altitude Han biomarkers. Unlike permanent Tibetan residents on the Qinghai-Tibet Plateau, who tend to have stable gut microbiota, the gut microbiota of the Han population changes with the duration of their residence on the plateau.^23^ Like seasonal gut microbiota changes among hunter-gatherers,^31^ gut microbiota remodeling may occur to adapt to changing environments. The plateau Han samples used in this study included people living there for three months to more than ten years. As the gut microbiota of the Han population varies with the time spent on a plateau, the performance of the training model is directly affected. Unfortunately, it is still unknown how the gut microbiota of the population that briefly lived on the plateau changed and when it stabilized. However, this would be a new concern for future research on high-altitude adaptation.

### Gut microbiota and altitude adaptation

Currently, research on the adaptation of the Tibetan Plateau population has focused on the relationship between genetic variations and high-altitude adaptation.^32,33^ However, there are few reports on the causal relationship between high-altitude adaptation and gut microbiota. High-altitude polycythemia, a prominent ailment, can lead to secondary thrombosis, bleeding, extensive organ damage, and sleep disturbances, posing a health risk to high-altitude populations.^34^ We found that some biomarkers in plateau Tibetan populations may help plateau Tibetan populations resist polycythemia as some OTUs belonging to *Prevotella* 9, *Eubacterium oxidoreducens*, Ruminococcaceae UCG005, Ruminococcaceae NK4A214. High-altitude diseases are more prevalent among migrants in high-altitude regions than among high-altitude Tibetan populations.^35^ Gut microbiota in Tibetan people may be one of the reasons why plateau Tibetans can better adapt to the plateau environment. Consequently, we believe that the gut microbiota may help Tibetan populations adapt to high-altitude environments more efficiently than Han populations. Although we used Mendelian randomization to investigate the relationship between gut microbiota and polycythemia, direct data on microbiota colonization are still lacking. Therefore, our future research will center on isolating gut microbiota and elucidating their functions. Notably, our recent study found that butyrate improved high-altitude polycythemia (unpublished data). This implies that certain butyrate-producing bacteria may be involved in the development of high-altitude polycythemia.

Furthermore, extensive cohort studies have revealed associations between host genetic single-nucleotide polymorphisms (SNPs) and the gut microbiota. Turpin et al.^36^ discovered that approximately one-third of fecal bacterial taxa display heritability, identifying 58 SNPs linked to the abundance of 33 bacterial taxa in 1098 participants. Hughes et al.^37^ found strong evidence for associations between host SNPs and 11 gut microbiota. Despite numerous SNPs being associated with high-altitude adaptation in Tibetan populations, their relationship with the gut microbiota remains unclear. Nonetheless, we view this as a stimulating avenue of research that could deepen our understanding of the genetic evolutionary interactions between gut microbiota, host genes, and high-altitude adaptation. This is a problem that we plan to investigate.

Most gut microbiota biomarkers in high-altitude populations were associated with the Ruminococcaceae and Lachnospiraceae families, represented explicitly by OTUs from the genera *Roseburia*, *Faecalibacterium*, *Dorea*, and *Coprococcus* 3. These gut microbiota biomarkers are important for overall health and are significant producers of SCFA.^19^ *Roseburia intestinalis* ^38^ and *Faecalibacterium prausnitzii* ^39,40^ have been extensively documented for their beneficial effects on various conditions, including digestive diseases, autoimmune diseases, metabolic disorders, and tumors. Moreover, we found a higher concentration of butyrate-producing bacteria in the high-altitude populations. Although there are no specific studies on SCFA content in the gut of high-altitude populations, it has been observed that the levels of acetate, butyrate, and total SCFA in the gastrointestinal tract of the Qinghai-Tibet Plateau pika are significantly higher than those in the Inner Daurian pikas.^41^ Under the same feeding conditions, Tibetan sheep produced higher concentrations of total SCFA, acetate, butyrate, and isovalerate, but lower concentrations of propionate than small-tailed Han sheep.^42^ These findings suggest that butyrate plays an essential role in the gut of high-altitude humans and animals. We found that butyrate treatment in rats resulted in a significant reduction in intestinal damage caused by the high-altitude environment in both the small and large intestines, as well as improved intestinal barrier function.

Moreover, we think butyrate may reduce intestinal damage by decreasing HIF-1α expression in hypoxic cells. However, recent studies on colitis appear to contradict this viewpoint. According to one report, using HIF-1α^ΔIEC^ mice, butyrate no longer alleviates DSS-induced colitis.^43^ Another study demonstrated that butyrate’s protective effect on Clostridium difficile-induced colitis disappears in HIF-1α^ΔIEC^ mice.^44^ Butyrate inhibits PHD2 in an HIF-1α-dependent manner, preserving intestinal barrier integrity and function.^45^ This highlights the importance of HIF-1α stability in mitigating colitis. However, a contradictory study found that hypoxia exacerbated both the symptoms and pathological damage in mice with Citrobacter rodentium-induced colitis. In these mice, HIF-1α significantly decreases under normoxic conditions but increases during hypoxic conditions. HIF-1α stability does not protect against colitis, as it activates the TLR4/NF-κB inflammatory pathway in high-altitude environments.^46^ Similarly, in SMAO-induced intestinal injury, HIF-1 activation is correlated with impaired intestinal barrier function and mucosal inflammation.^47^ Emodin inhibits the HIF-1α and NF-κB signaling pathways, alleviating LPS- and HR-induced intestinal epithelial barrier dysfunction.^48^ Propionic acid can prevent burn-related intestinal barrier damage by inhibiting HIF-1α.^49^ Hence, HIF-1α stability is advantageous in specific circumstances, but HIF-1α overexpression may compromise intestinal barrier stability in high-altitude settings. In a mice study, conditional HIF-1α mutant mice showed protected intestinal permeability under hypoxic conditions, suggesting HIF-1α involvement in heightened permeability during hypoxia.^50^ Our findings indicate that, whereas butyrate diminishes HIF-1α expression, it enhances the intestinal barrier protein occludin expression. Consequently, we propose that HIF-1α may play diverse roles in intestinal health. Maintaining physiological hypoxia and stabilizing HIF-1α under normoxic conditions promotes intestinal health, with butyrate aiding this process.^43,44^ Excessive HIF-1α expression in hypoxic conditions at high altitudes may increase intestinal inflammation and damage. However, butyrate may regulate HIF-1α to maintain steady levels, improving intestinal health.

### Butyrate and gene-blunting

Tibetan show delayed and weakened responses to hypoxia in the whole-genome expression pattern of oxygen-deprived cells in the Tibetan compared with Han,^20^ indicating a blunting effect. Based on RNA-seq data analysis of Tibetan and Han HUVEC, HIF-1 and glycolysis pathways were discovered significant enrichment in hypoxia. However, Tibetans hypoxia HUVEC exhibit smaller magnitudes of gene changes in the glycolysis and HIF-1 pathway. Interestingly, these results are similar to trends observed in certain high-altitude animals. In low-oxygen conditions, plateau pikas showed the increase in HIF-1α and VEGF in plateau pikas was significantly lower than that in SD rats in their skeletal muscles.^51^ A comparison of relevant gene expression in the livers of Qinghai-Tibet Plateau mammals and low-altitude mice after hypoxia showed that low-altitude mice displayed increased sensitivity of HIF-1α to oxygen compared to the high-altitude species *Ochotona curzoniae* and *Microtus oeconomus* .^52^ Similar results were obtained during chicken egg incubation. On days 12 and 15 of incubation at high altitudes, Tibetan chicken embryos had significantly lower blood lactate levels and LDH activity (breast muscle and heart) than dwarf chicken embryo. Additionally, the activity of succinic dehydrogenase, a TCA enzyme, was significantly higher in the heart tissue of high-altitude Tibetan chickens than that of high-altitude dwarf chickens, which may mean that Tibetan chickens have a respiratory advantage over Dwarf chickens living at high altitudes.^53^ Notably, our examination of hypoxia-stimulated cells treated with butyrate indicated significant reductions in key glycolytic pathway genes (HK2 and PDK1) transcription levels and lower LDHA enzyme activity and lactate production. Furthermore, butyrate has also been found to significantly improve the average movement time of mice and raise the mRNA expression levels of genes related to mitochondrial biogenesis in the gastrocnemius muscle.^54^ This suggests that butyrate may help hypoxic cells undergo blunting in energy metabolism, similar to Tibetan cells’ adaptation to a hypoxic environment.

**Figure 9.**
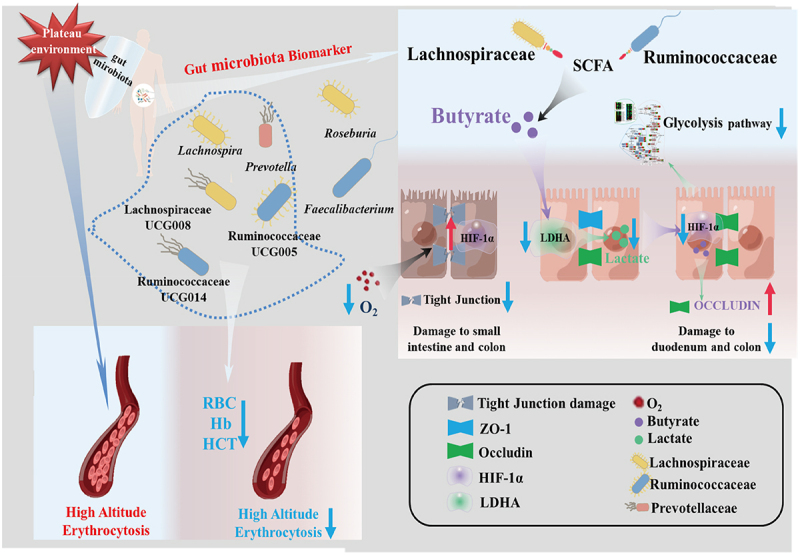
The potential mechanism of gut microbiota biomarkers to help maintain human health at the plateau.

As previously mentioned, the indigenous inhabitants of the Qinghai-Tibet Plateau rely on adaptive gene mutations, such as *EGLN*1, to help them adjust to high-altitude environments. *EGLN*1, an adaptive mutation gene found in Tibetans, encodes the PHD2 protein, which regulates the hydroxylation and degradation of HIF-α subunits through hydroxylation modification.^55,56^ In hypoxic settings, the *EGLN*1 [Asp4Glu; Cys127Ser] variant in Tibetans more actively downregulates HIFα subunits than the wild type.^11^ Although research on HIF-1α in the adaptation of plateau populations is currently limited, our results confirm the downregulation of glycolysis pathway genes after butyrate treatment, paralleling previously reported gene changes in specific plateau species. Thus, based on our findings and previous reports, we propose a speculative model of gut microbiota and high-altitude adaptation. We suggest that during the initial stages of acclimatization to high altitudes, the high-altitude environment causes intestinal damage, disruption of the intestinal barrier, and dysbiosis of the gut microbiota, exacerbating intestinal damage. As high-altitude adaptation progresses, the gut microbiota gradually stabilizes, gut microbiota biomarkers in plateau populations become enriched, and butyrate levels in the gut rise, minimizing the damage to the intestinal barrier caused by high altitude. Butyrate downregulates HIF-1α to achieve a relatively stable level to regulate glycolysis-related genes. Consequently, glycolysis in the gut is reduced, LDHA activity is directly inhibited, lactate production is decreased, oxygen utilization improves, and gut health is preserved (Figure 9). However, the exact mechanism by which butyrate decreases HIF-1α levels is unknown. Previous studies have demonstrated that lactate from adipose tissue can stabilize HIF-1α in macrophages via lactate binding directly to the catalytic domain of PHD2.^27^ Moreover, we found exogenous lactate helps stabilize HIF-1α in hypoxic cells. Thus, we hypothesize that butyrate reduces lactate production while lactate down-regulated the stability of HIF-1α. However, additional experimental data are still required to validate specific process, and we will be investigated in future studies, to provide a more comprehensive explanation.,

The high-altitude hypoxic environment can be detrimental to the body. On the one hand, reduced oxygen levels lead to high-altitude polycythemia, but specific gut microbiota biomarkers can dull the erythrocyte production response, aiding adaptation to the high-altitude environment. On the other hand, the high-altitude hypoxic environment can lead to intestinal and intestinal barrier damage and gut microbiota imbalance. Gut microbiota biomarkers in people living in plateau regions protect against intestinal damage caused by the harsh environment. Butyrate produced by gut microbiota biomarkers may reduce lactic acid build up in the intestinal tract and decrease the overexpression of HIF-1α, which is caused by hypoxia at high altitudes, by inhibiting LDHA activity. Inhibiting the downstream target gene of HIF-1 can help the host better adapt to the plateau environment, maintain the stability of the intestinal barrier, and reduce intestinal damage. RBC: Erythrocyte count; Hb: Hemoglobin concentration; HCT: Hematocrit. The figure is drawn using Figdraw. (https://www.figdraw.com/).

While we were unable to obtain a pure culture of gut microbiota biomarkers, we recommend exploring this possibility through microbial culture technology in the future to further test their function in high-altitude adaptation. In conclusion, we identified gut microbiota biomarkers in plateau populations, primarily Ruminococcaceae and Lachnospiraceae, and found that butyrate can mitigate intestinal damage caused by high-altitude conditions and that butyrate produced by gut microbiota may be associated with hypoxia-induced gene blunting in high-altitude adaptation. Our findings open new avenues of inquiry into the role of the gut microbiota in host adaptation to high-altitude environments.

## Materials and methods

### Public data collection and description

A total of 668 original 16S rRNA sequencing data of Tibetan and Han populations at the Qinghai-Tibetan Plateau and low-altitudes in these six studies were then retrieved from SRA (Sequence Read Archive), ENA (European Nucleotide Archive) and GSA (Genome Sequence Archive) and their accessible raw data identification numbers were obtained, which were PRJCA001483 from Zhilong Jia et al.,^23^ PRJNA381333 from Daoliang Lan et al.,^57^ PRJNA507100 from Yixuan Liu et al.,^58^ PRJNA665364 from Yue Xiao et al.,^59^ PRJNA699380 from Yulan Ma et al.^60^ and PRJCA002832 from Tian Liang et al..^61^ The samples were divided into four groups according to their origins, LH (low altitude Han group), HH (high altitude Han group), LT (low altitude Tibetan group) and HT (high altitude Tibetan group). The details of all raw data (age, sex and Body Mass Index[BMI]) are provided in Figure 1 and Supplementary Table S1. The hosts of these samples are free from known diseases and have not used antibiotics in the three months preceding sampling. Given the distinct dietary habits between Tibetans and Hans, low-altitude Tibetans in Study 6 exhibit similar dietary patterns to Hans. Consequently, we compared LH_vs_LT to evaluate the correlation between differential gut microbiota and ethnicity and altitude. Furthermore, only Study 3 (HT) and Study 4 (LH) include data on the age and sex of the samples, whereas the remaining studies lack complete personal information. Furthermore, for model validation, the low altitude Han population in PRJNA578008 from Zeng et al.^62^ Supplementary Data 10 for details of the raw data.

### Volunteer recruitment and sample collection

Volunteer recruitment and sample collection were approved by Human Research Ethics Committee of China Agricultural University (Approval No. CAUHR-2019010). All participants were informed and aware of the study. A written consent was obtained from each participant. This study protocol is in agreement with the world medical association declaration of Helsinki (2008) and the Belmont Report. 40 high-altitude samples were collected of volunteers recruited from Lhasa, Nagqu, Qamdo, Yushu in Qinghai-Tibet Plateau. 4 low-altitude Han volunteers came from Guangyuan in Sichuan Province, Lvliang in Shanxi Province, and Lanzhou in Gansu Province, and had not been to the Qinghai-Tibet Plateau. All volunteers were free of diseases and had not taken antibiotics within the 3 months before sampling. The fecal samples were stored in fecal collection tubes with fecal preservation solution, then returned to the laboratory and stored at −80°C.

### 16S rRNA sequencing

Microbial community genomic DNA was extracted from fecal samples using the E.Z.N.A.® soil DNA Kit (Omega Bio-tek, Norcross, GA, U.S.) according to the manufacturer’s instructions. The hypervariable region V3-V4 of the bacterial 16S rRNA gene were amplified with primer pairs 338F (5’-ACTCCTACGGGAGGCAGCAG-3’) and 806 R(5’-GGACTACHVGGGTWTCTAAT-3’) by an ABI GeneAmp® 9700 PCR thermocycler (ABI, CA, USA).The PCR product was extracted from 2% agarose gel and purified using the AxyPrep DNA Gel Extraction Kit (Axygen Biosciences, Union City, CA, USA) according to the manufacturer’s instructions and quantified using Quantus™ Fluorometer (Promega, USA). Purified amplicons were pooled in equimolar and paired-end sequenced on an Illumina MiSeq PE300 platform/NovaSeq PE250 platform (Illumina, San Diego,USA) according to the standard protocols by the Majorbio Bio-Pharm Technology Co. Ltd. (Shanghai, China).

### Data processing

To combine diverse sequencing data across multiple studies and tackle the errors induced by PCR biases, we implemented the methods for processing data and approaches utilized by Jun Yuan et al..^63^ The raw data were processed using the join_paired_ends.py script in QIIME (V.1.9.0)^64^ to merge the paired-end reads. The split_libraries_fastq.py script was then used to trim the reads to a minimum Phred score of 20. Next, the VSEARCH (V.2.23.0)^65^ software was used with the usearch global algorithm to map all sequences to the Silva database (V.132 based on a 97% similarity threshold. Only OTUs present in at least five studies and appearing in at least one-third of the samples were retained for subsequent analysis. The Alpha diversity of samples was assessed using QIIME2 (V.2020.8),^66^ computing the Shannon, observed OTUs, and evenness indices. For evaluating beta diversity, the Bray-Curtis Dissimilarity was calculated with the “vegan” package (V.2.5–7) in R (V.4.0.4).

### Meta-analysis batch effect adjustment

To regulate batch effects in a meta-analysis, we used the blocked Wilcoxon Rank sum test and tools that specialize in modulating batch effects in the microbiome ConQuR the samples implemented in the R (V.4.0.4) “coin” package and “ConQuR” package, respectively.

### Identification and model construction of gut microbiota biomarkers

First, OTU with significant differences in LH_vs_HH, LH_vs_HT and LH_vs_LT were evaluated by the Wilcoxon Rank sum test using the “stats” package in R (V.4.0.4). These significantly different OTUs (p.adj <0.05) were screened several times using the “Boruta (V.7.0.0)” package. Finally, the “RandomForest (V.4.6–14)” package was used to evaluate the optimal number of markers to determine the gut microbiota biomarkers in LH_vs_HH, LH_vs_HT and LH_vs_LT, respectively. We aggregated all the samples to construct a 10-fold cross-validation RF model. We repeated the process ten times to determine our analysis’s optimal number of gut microbiota biomarkers. Then, these gut microbiota biomarkers were used in R (V.4.0.4) to construct a RandomForest (RF) model, Robust Linear Model (RLM) and Support Vector Machines (SVM) model using “randomForest (V.4.6–14)” package, “Caret (V.6.0–90)” package and “KernLab (V.0.9–29)” package, respectively. In the process of model construction, 80% of the samples were used as a training set, and 20% of the samples were used as a test set. The model was trained by the method of 10-fold cross-validation.Finally, AUC, accuracy, sensitivity, specificity, precision, and F1 score were used to evaluate the performance of the model. The rationality of features extracted as gut microbiota markers in high and low altitude populations were evaluated.

The above-mentioned methods were consistently used for feature extraction, model construction and performance evaluation methods at phylum, class, order, family, genus and species levels

### Gut microbiota biomarkers and model evaluation

The universality of identified gut microbiota biomarkers and gut microbiota classifier for high-altitude population was evaluated. According to the method used by Yuanqi Wu et al.,^18^ study-to-study transfer validation and Leave-one-dataset-out (LODO) validation were adopted for evaluation. In the study-to-study transfer validation, all samples in each single study were used as the training set to construct an RF classification model for gut microbiota markers, and samples from all other studies were used as test sets for evaluation. Meanwhile, training samples were used as test sets for internal assessment of the constructed classification model. AUC was used as the evaluation index. In LODO validation, a single study was used as the test set, and all the remaining samples were used as the training set to construct an RF classification model for gut microbiota markers. Due to the requirements for participating in Study-to-Study transfer and LODO validation, it is crucial to ensure that each individual study incorporates a minimum of two distinct groups. However, Study 3 solely includes the HT group, while Study 4 exclusively comprises the LH group. To meet the criteria for Study-to-Study transfer and LODO validation, and given the absence of LH group data in Study 2, we amalgamated Study 2 and Study 4, forming Study 24. Moreover, as Study 1 and Study 5 encompass comprehensive datasets for LH, HH, and HT, they were not amalgamated with Study 6. Ultimately, Study 3 and Study 6 were amalgamated to create Study 36 for subsequent Study-to-Study transfer and LODO validation.

Performance of the gut microbiota markers was evaluated. Five groups of different combinations of OTUs were used, including (1) top gut microbiota marker, (2) all gut microbiota markers, (3) OTUs with significant differences and (4) all OTUs. Then, the RF classifier model was constructed according to the combination of the OTUs, and the model’s performance was evaluated using LODO validation.

To identify co-biomarkers of the high-altitude population, we consider OTU, which meets the following two conditions, as co-biomarkers of the high-altitude population. (1) Gut microbiota biomarker belonging to LH_vs_HH or LH_vs_HT; (2) The relative abundance of Gut microbiota biomarker was significantly higher in HH and HT than in LH. Then, a classification model was constructed using the identified gut microbiota co-biomarkers of the high-altitude population with the RF method, and the ability of these OTUs as co-biomarkers to distinguish between high and low-altitude populations was evaluated. Finally, all samples (except LT) were used as the training set, and a batch of independent samples was introduced as the test set to reevaluate these co-biomarkers. The LT samples were used to assess whether these biomarkers were associated with ethnicity. If the relative abundance of these biomarkers is also significantly higher in LT compared with LH, it is suggested that these markers enriched in high-altitude populations may be ethnically related.

### Two-sample Mendelian randomisation analysis

The exposed GWAS data is sourced from MiBioGen,^67^ a large-scale multi-ethnic GWAS coordinating 24 cohorts from 11 countries, 16S ribosomal RNA gene sequencing profiles from 18,340 participants involving 211 gut microbiota taxonomic groups. Outcome data are derived from traits curated by the IEU Open GWAS project(https://gwas.mrcieu.ac.uk/), with Supplementary Data 11 providing detailed information on these datasets. SNP variants significantly associated with the gut microbiome were selected as instrumental variables (IVs), with an IV threshold set at *p*-value <1 × 10–5.^68^ Linkage disequilibrium (LD) between SNPs was assessed using a clumping process (R2 < 0.01 and clumping distance = 500 kb).^69^ The primary assessment method was Inverse Variance Weighted, supplemented by MR Egger, Weighted Median, Simple Mode, and Weighted Mode, with results deemed credible when five methods showed consistent causal directions. MR-Egger and MR-PRESSO methods detected potential horizontal pleiotropic effects (MR-PRESSO global test *p*-value > 0.05 and MR-Egger regression *p*-value > 0.05 indicating no horizontal pleiotropy). Heterogeneity was assessed using Q statistics, with a *p*-value < 0.05 signifying the presence of heterogeneity. The MR analysis used the R package TwoSampleMR (V.0.5.6).

### Analysis of SCFA-producing gut microbiota

The propionate and butyrate production capacity of the gut microbiota was assessed following the approach outlined by Berenike Kircher et al.^25^ SCFA pathway predictions were conducted using the PICRUSt2 algorithm (v2.4.1)^70^ by inserting sequences into the reference tree (place_seqs.py) followed by hidden-state predictions (hsp.py).

### Data mining of RNA sequencing data

The data was retrieved from the GEO database with accession number GSE145774, comprising RNA sequencing data from both Tibetan and Han Chinese HUVECs across multiple time points under both normoxic and hypoxic conditions. Differential gene analysis was executed utilizing the GEO2R tool available within the GEO database. We employed the R package ClusterProfiler (version 4.8.1) for KEGG pathway enrichment analysis, and *p*-values underwent correction using the FDR.

### Rat

All experiments were conducted in accordance with the requirements of China Agricultural University Laboratory Animal Welfare and Animal Experiment Ethics Committee (Approval No. Aw21502202-3-1). Twenty 3-week-old male SD rats were purchased from the Beijing Huafukang Biotechnology Co., LTD. Twenty-four SD rats were randomly divided into four groups, namely plain group, high altitude group, propionate group and butyrate group. Then, the propionate group and butyrate group were gavaged with sodium propionate (Yuanye, #S30148) and sodium butyrate (Yuanye, #S30223) once a day for 24 consecutive days, respectively. The standard gavage of 50 mg/100 g in rats^16,71^ was dissolved with PBS buffer solution (the amount was 1 mL per 100 g of mice). The plain group and high-altitude group were given an equal volume of PBS buffer once a day for 24 consecutive days. Before entering the high-altitude region, the rats were raised in the Experimental Animal Center of the State Key Laboratory of Agricultural Biotechnology, China Agricultural University, Beijing, China (altitude: 50 m).

### High-altitude model establishment and histological examination

After 24 days of continuous intragastric administration, rats in the high-altitude group, the propionate group and the butyrate group were immediately transported to the Nagqu region of Tibet (4500 m) by air. On the 6th day of their arrival in Nagqu, rats were treated with cervical dislocation, and about 2 cm of their duodenum, jejunum, ileum and colon immediately taken and placed in 4% paraformaldehyde (PFA) (Sigma, #P6148) at 4°C for preservation.

After fixing with 4% of PFA for 24 h, the tissue was dehydrated with gradient ethanol and then embedded with paraffin. The paraffin tissue was cut into 5 μm thick sections and stained with the hematoxylin and eosin (HE) staining kit (Abcam, # AB245880). The intestinal morphology was observed by microscope, and the length of villi and crypt depth were measured by ImageJ.

To evaluate the villi length and crypt depth of small intestine tissue, 3 sections were taken from each tissue and 3 different fields were randomly selected for each section. A total of 8 villi and crypts were selected for statistical analysis under each field. Therefore, a total of 72 villi and crypts of each tissue were measured.

### Immunohistochemical staining

Immunohistochemistry was used to stain the occludin antigen in the paraffin sections of the duodenum and the ZO-1 and occludin antigens in the paraffin sections of the colon. After blocking endogenous peroxidase, sections were prepared at 4°C with rabbit polyclonal antibody (ZO-1, 1:250, Proteintech, # 21773–1-AP; Occludin, 1:250, Proteintech, # 27260–1-A) incubated together overnight. Next, according to the instructions of the manufacturer of the Universal two-step test Kit (ZSJB-BIO,#PV-9000), the tissues were then colored by 3′, 3-diaminobenzidine tetrahydrochloride (DAB; ZSJB-BIO, # ZLI-9018). The slices were then stained with hematoxylin and fixed. Three regions of each sample were randomly selected to evaluate positive signals. Then the Mean integral optical density (Mean IOD) of the tissue was determined by Image Plus Pro 6.

### The contents of butyric acid and lactic acid were determined by HPLC

To determine the content of butyric acid in rat stool, weigh 0.2–0.3 g rat stool, add the corresponding ultra-pure water according to the concentration of 10 ml/g, shake and mix, centrifuge at a low temperature of 12000 rpm for 20 min, and then use a 0.45um filter to collect the filtrate for the detection of butyric acid content. To determine the lactic acid content in the cells. After cell lysis, part of the cell was used for protein concentration determination, and the other part use a 0.45um filter to collect the filtrate for the detection of lactic acid content.

HPLC determination was performed using a MARS MOA chromatographic column and an RID detector. The mobile phase consisted of 2.5 mM H2SO4 with a flow rate of 0.6 mL/min. The column temperature was set at 60°C, and the injection volume was 20 μL. The peak retention time of the analyte was determined based on the standard substance.

### Cell culture and treatments

Human normal colonic epithelial cells NCM460 were donated by Professor Yu Zhengquan from China Agricultural University and cultured in RPMI1640（Gibco, #C11875500CP） medium containing 10% FBS (Newzerum, # FBS-S500) and 1% sp. After the cells were cultured to 100% confluence, the cells were treated with the corresponding concentrations of sodium butyrate (Yuanye, #S30223) and CoCl_2_(Sigma, # C8661) and continued to be cultured in a medium containing 1% FBS for 12 or 24 h.

### Total RNA isolation and real-time PCR

Total RNA was extracted by Total RNA Extract Reagent (Coolaber，#RE600) and reverse transcribed into first strand cDNA using the RT Reagent Kit (Genstar, #A234–10). Real-time PCR reactions were performed using 2×RealStar Power SYBR qPCR Mix (Low ROX) (Genstar, #A314). The qPCR procedure was as follows: pre-denaturation at 95°C for 10 min, 40 cycles of denaturation at 95°C for 15 s and annealing at 60°C or 62°C for 60 s.The 2−^ΔΔCt^ values between candidate genes and β-Actin or 16s Ct values were calculated by qPCR analysis. The primer sequences and annealing temperature used are listed in Supplementary Table S6.

### Western blot

Total protein was extracted from cultured cells using RIPA Lysis Buffer (Beyotime, #P0013B) and the protein concentration was determined by BCA kit (Genstar, #E162). The total protein was loaded onto a sodium dodecyl sulfate-polyacrylamide gel and ran at 120 volts for 2 hours. After electrophoresis, the proteins were transferred to a polyvinylidene fluoride membrane and treated with 5% skim milk TBST (50 mM Tris; 150 mM NaCl; 0.05% Tween 20) closed for one h. The PVDF membrane was then incubated at 4°C overnight with primary antibody and at room temperature with enzyme-labeled secondary antibody for 1 hour. Super-sensitive ECL chemiluminescent substrate (Biosharp, #BL520A) was used to reveal protein bands. The antibodies used are: ZO-1 (1:6000, Proteintech, # 21773–1-AP), HIF-1α (1: 6000, Proteintech, #20960–1-AP), Occludin(1:6000, Proteintech, # 27260–1-AP), alpha-tubulin (1:2000, Proteintech, #11224–1-AP), Goat Anti-Rabbit IgG (H + L)-HRP Conjugate(1:4000, Bio-Rad, #1706515). ImageJ is used for grayscale measurement.

### Determination of LDHA activity

The experimental principle for determining specific activity was that lactate dehydrogenase catalyzed the mutual conversion of lactic acid and pyruvate. The reaction system was determined by referring to the LDH-specific activity determination method recommended by Worthington (http://www.worthington-biochem.com). The reaction system was as follows: 0.2 M Tris-HCl (pH = 7.3) buffer 2.8 mL, 6.6 mmol/L NADH 0.1 mL, 30 mmol/L sodium pyruvate 0.1 mL, total protein 100ul. A spectrophotometer measured the change of light absorption value at 340 nm. LDHA relative activity =△_A340_/total protein concentration.

### Statistical analysis

The Wilcoxon Rank sum test was used to test the significance of differences between samples in biological informatics analysis. The t-test was used to determine the differences between groups in animal and cell experiments. In the Beta diversity analysis, the permutational multivariate analysis of variance (PERMANOVA) was performed on the Bray-Curtis dissimilarity matrix to determine whether the Beta diversity was the same between different samples. The plots in this study were created using the “ggplot2 (V.3.3.5)” package in R (V.4.0.4).

## Supplementary Material

Supplemental Material

## Availability of supporting data and code

The raw 16S rRNA gene sequencing data are available from the Sequence Read Archive (SRA) (https://www.ncbi.nlm.nih.gov/sra) 、European Nucleotide Archive (ENA) (https://www.ncbi.nlm.nih.gov/) and Genome Sequence Archive(GSA) (https://ngdc.cncb.ac.cn/gsa/), with project ID: PRJCA001483, PRJNA381333, PRJNA507100, PRJNA665364, PRJNA699380, PRJCA002832 and PRJNA57800. The remaining data are available within the Article, Supplementary Information. The codes and scripts are available at https://github.com/Yangjsh1999/plateau.
