# Supplementary material for: Meta-analysis identifying gut microbial biomarkers of Qinghai-Tibet Plateau populations and the functionality of microbiota-derived butyrate in high-altitude adaptation
Source: Gut Microbes. 2024 May 7;16(1):2350151. doi: 10.1080/19490976.2024.2350151 (PMC11086029; doi:10.1080/19490976.2024.2350151)

**Meta-analysis identifying gut microbial biomarkers of Qinghai-Tibet Plateau populations and the functionality of microbiota-derived butyrate in high-altitude adaptation**

Hongwen Zhao^a^, Longjie Sun^a^, Jiali Liu^a^, Bin Shi^b^, Yaopeng Zhang^a^, Ci-ren Qu-zong^c,d^, Tsechoe Dorji^c^, Tieyu Wang^e^, Hongli Yuan^a^, Jinshui Yang^a^*

*To whom correspondence should be addressed. E-mail: yangjsh1999@cau.edu.cn

This file includes:

Supplementary Fig. 1 to 16

Supplementary Table 1 to 6

**
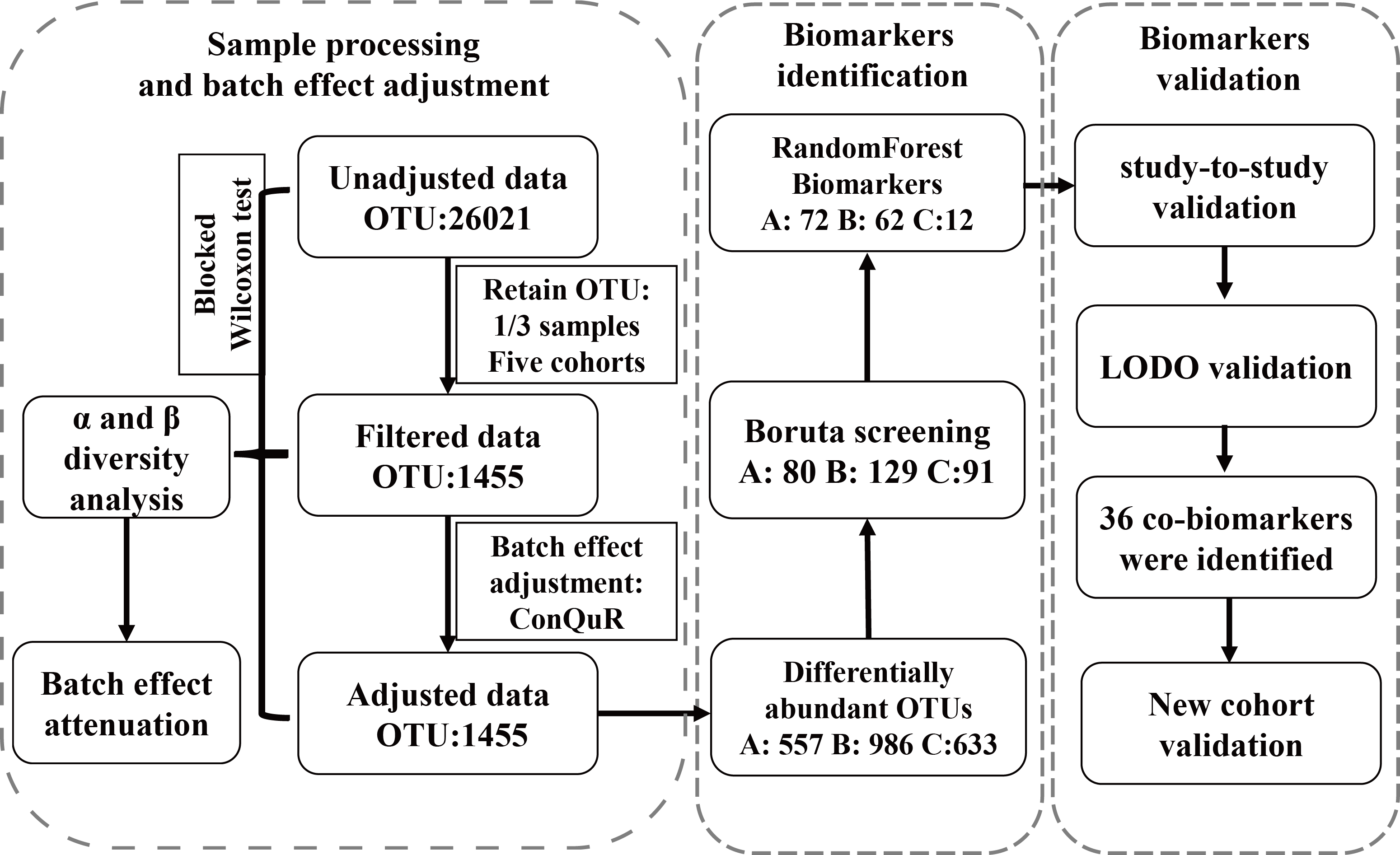
****Supplementary Fig. 1** Microbiome analysis flowchart. Sample processing and batch effect adjustment: Initially, 26,021 OTUs were derived from the raw data. Subsequent α and β diversity analyses and the two-sided blocked Wilcoxon rank-sum test exposed batch effects across different batches. To mitigate this, the raw data underwent filtration to retain OTUs prevalent in over one-third of the samples and spanning at least five studies, resulting in the retention of 1,455 OTUs, representing 68.51% of total reads with an average of 33,494 reads per sample. Despite these measures, α and β diversity analyses and the two-sided blocked Wilcoxon rank-sum test continued to signal the presence of batch effects. Consequently, ConQuR was employed to rectify these issues. Biomarker identification was then carried out on the adjusted data, using a sequential approach involving the Wilcoxon rank-sum test (*p* < 0.05), Boruta, and Random Forest methods, leading to the identification of gut microbiota biomarkers (LH_vs_HH: 72, LH_vs_HT: 62, LH_vs_LT: 12). Subsequently, Biomarker validation ensued, utilizing Study-to-Study transfer and LODO validation methods. Additionally, 36 gut microbiota co-biomarkers to the high-altitude population were discerned and validated using new cohorts. A: LH_vs_HH,
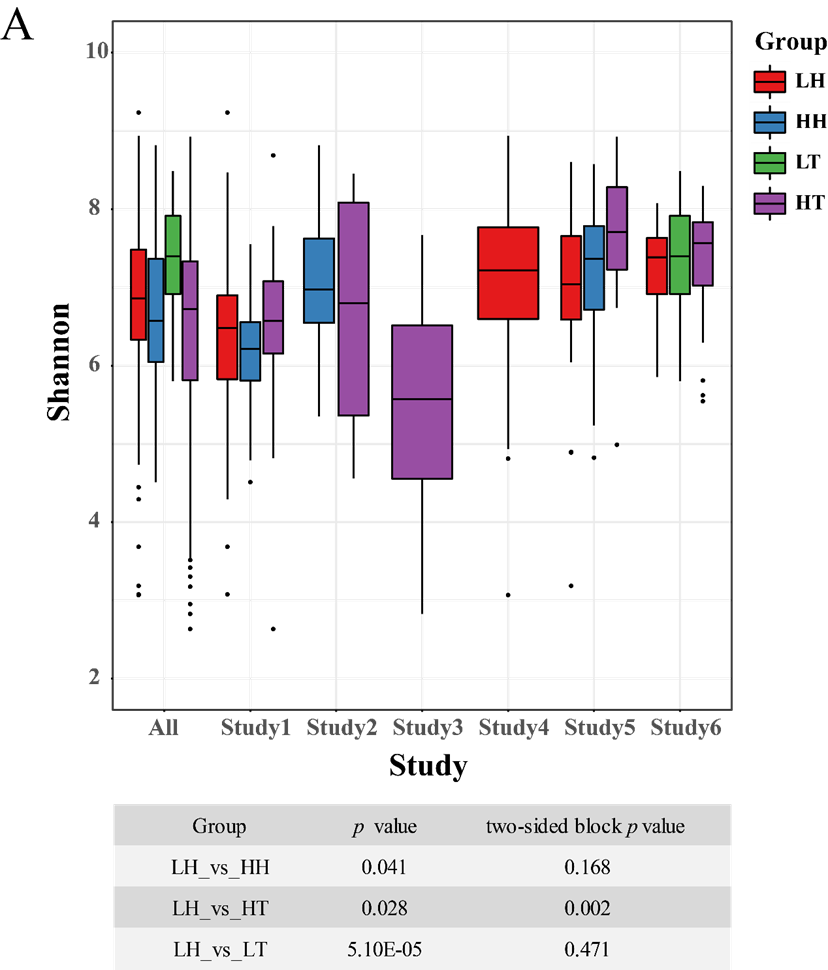
B: LH_vs_HT, C: LH_vs_LT.


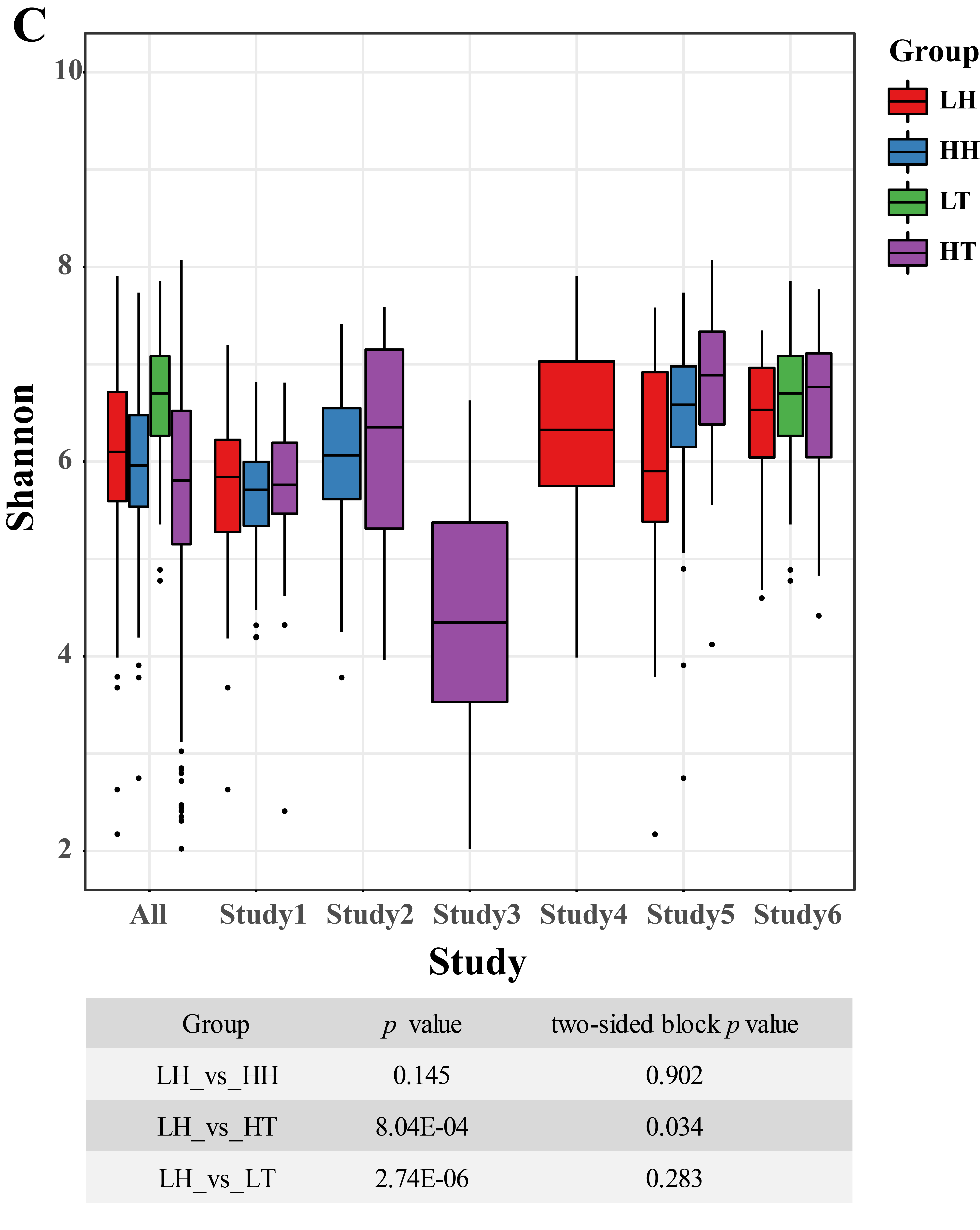

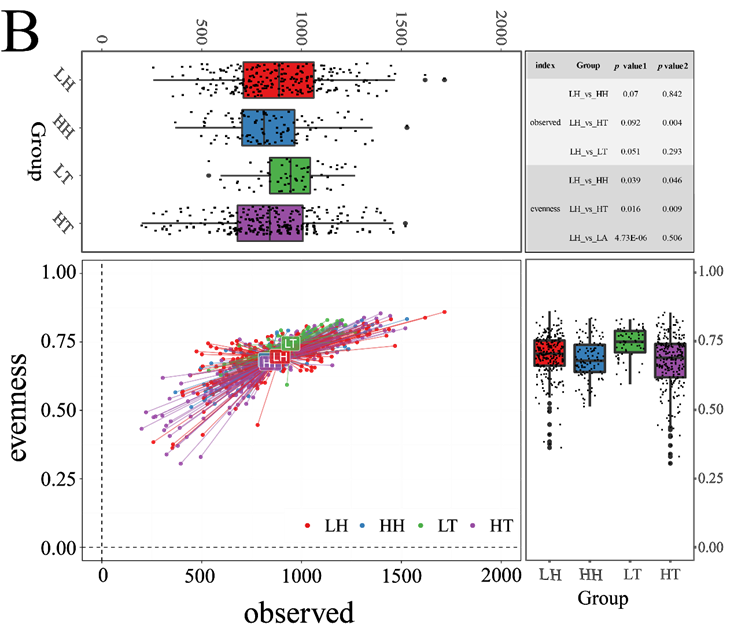


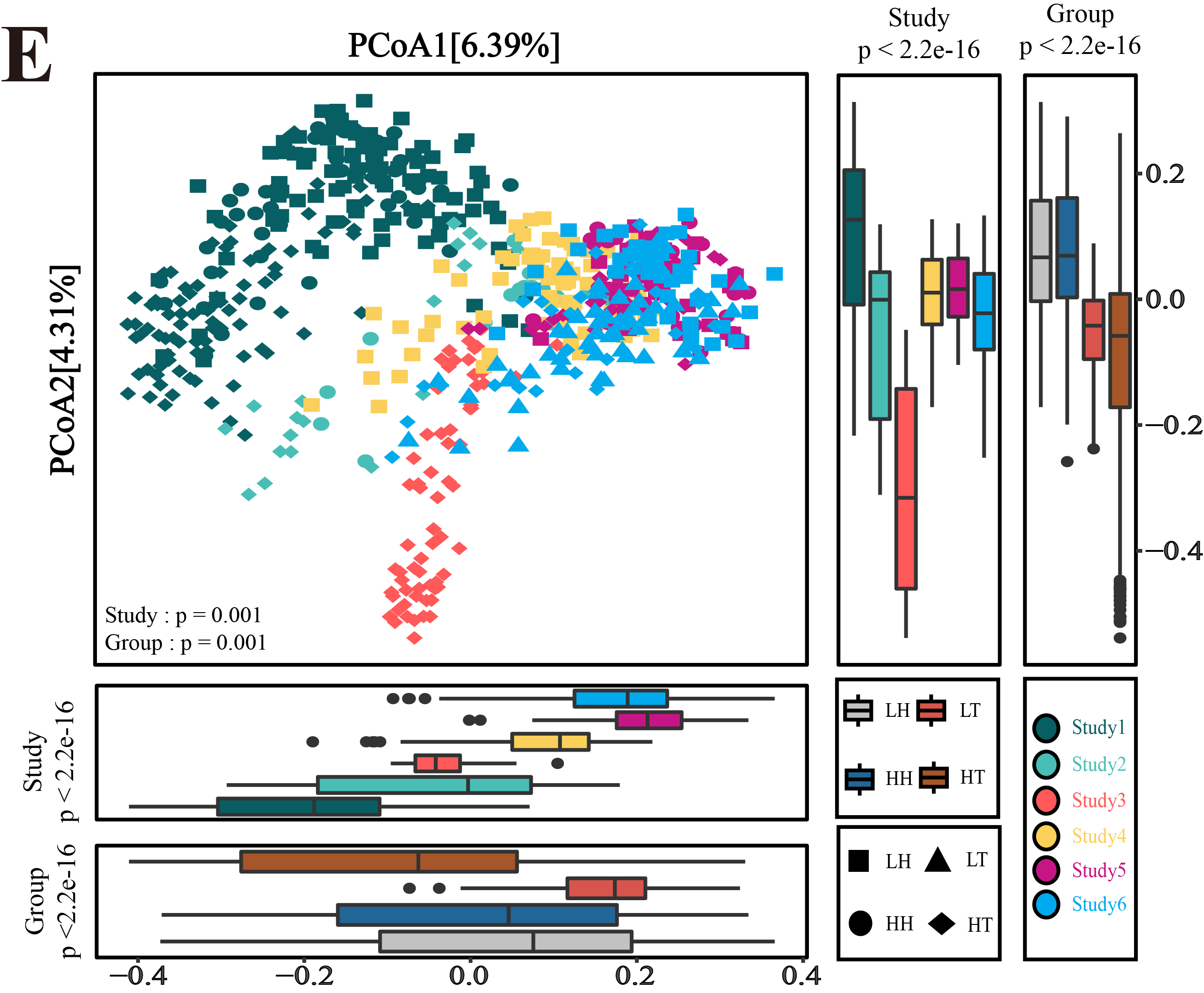

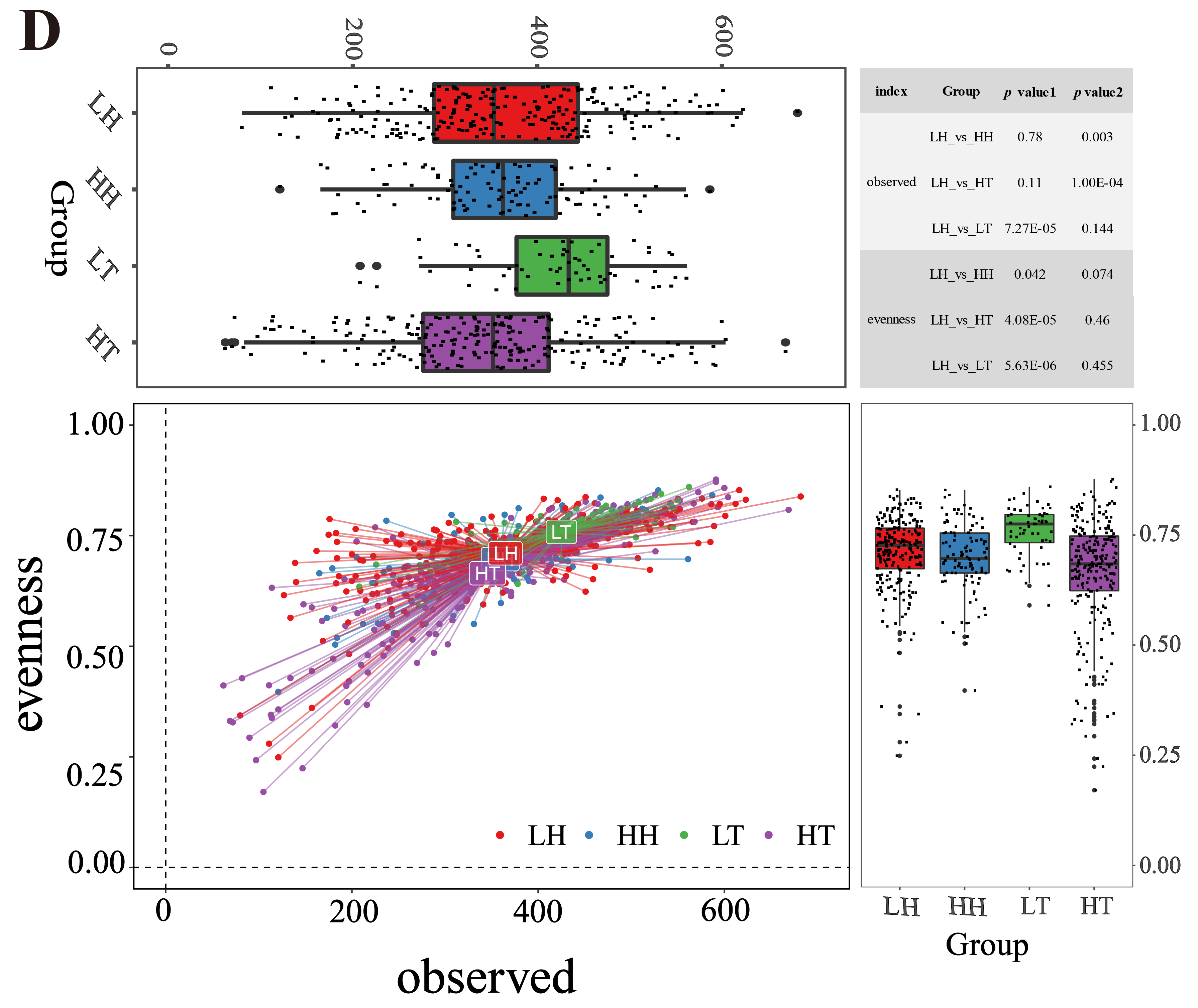


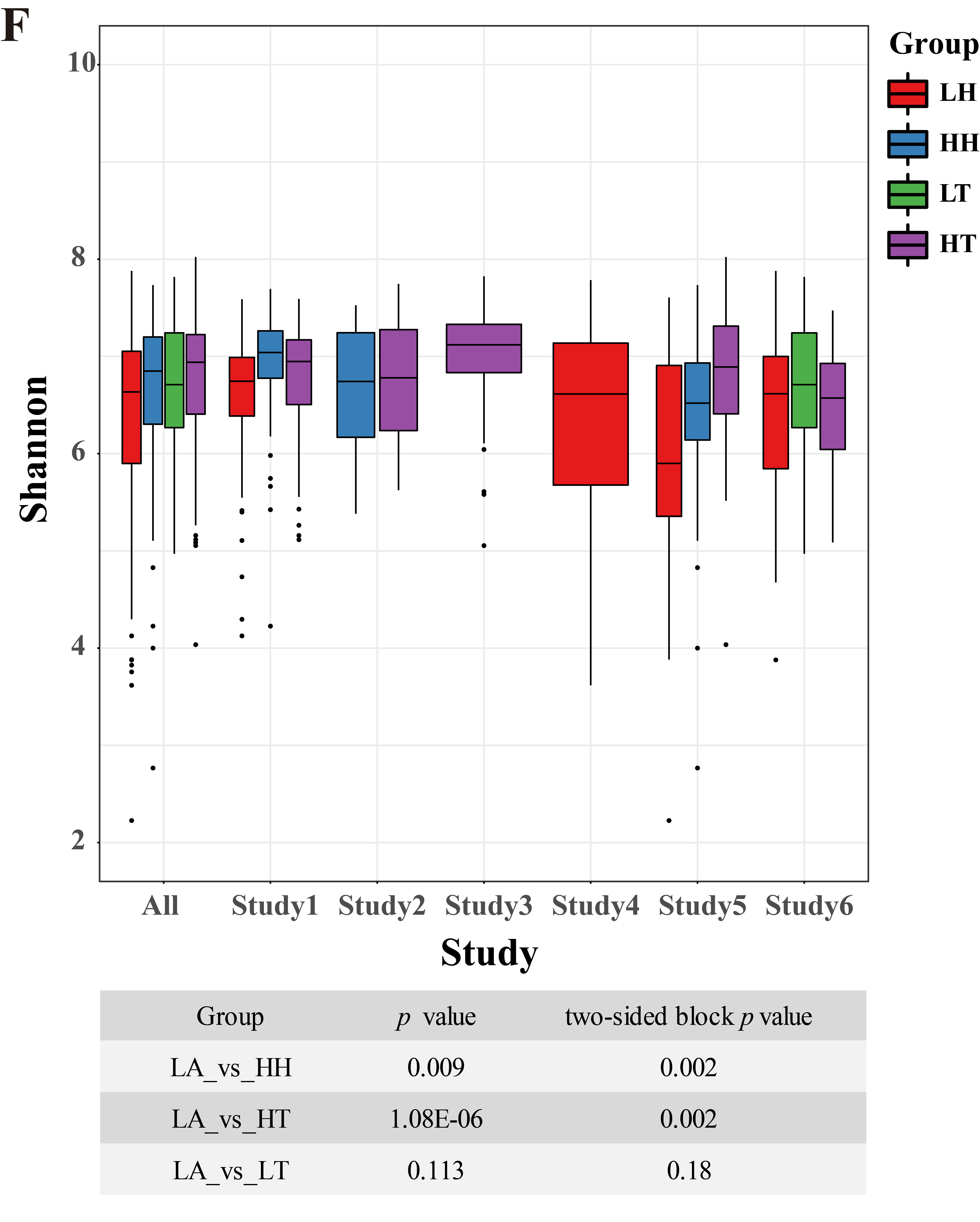
**Supplementary Fig. 2 Alpha and Beta diversity analysis before batch effect adjustment.**

**(A)** Shannon index (LH, n=243; HH, n = 105; LT, n=59; HT, n=240), an index of species richness and evenness. The *p* value in the table was from a Wilcoxon Rank sum test or a blocked Wilcoxon Rank sum test. **(B)** Richness index and evenness index scatter plot (LH, n=243; HH, n = 105; LT, n=59; HT, n=240). The box plot on the top indicates the observed index results of different groups, while the box plot on the right indicates the evenness index results of samples of other groups. The table on the top right shows the difference between groups in observed and evenness index compared with LH. *p* value 1 was from a two-sided Wilcoxon rank-sum test. *p* value 2 is was from a Wilcoxon Rank sum test. **(C)** Shannon index after filtering some OTUs processing (LH, n=243; HH, n = 105; LT, n=59; HT, n=240), an index of species richness and evenness. The *p* value in the table was from a Wilcoxon Rank sum test or a blocked Wilcoxon Rank sum test. **(D)** Richness index and evenness index scatter plot after filtering some OTUs processing (LH, n=243; HH, n = 105; LT, n=59; HT, n=240). The box plot on the top indicates the observed index results of different groups, while the box plot on the right indicates the evenness index results of samples of other groups. The table on the top right shows the difference between groups in observed and evenness index compared with LH. *p* value 1 was from a two-sided Wilcoxon rank-sum test. *p* value 2 is was from a Wilcoxon Rank sum test. **(E)** PCoA analysis of samples after filtering some OTUs processing (LH, n=243; HH, n = 105; LT, n=59; HT, n=240). Beta diversity was based on Bray-Curtis Dissimilarity. *p* value of the PCoA was from PERMANOVA (999 permutations). The *p* value of the boxplot was from the Kruskal test.

(F) Shannon index after filtering some OTUs and ConQuR processing (LH, n=243; HH, n = 105; LT, n=59; HT, n=240), an index of species richness and evenness. The *p* value in the table was from a Wilcoxon Rank sum test or a Wilcoxon Rank sum test.

**
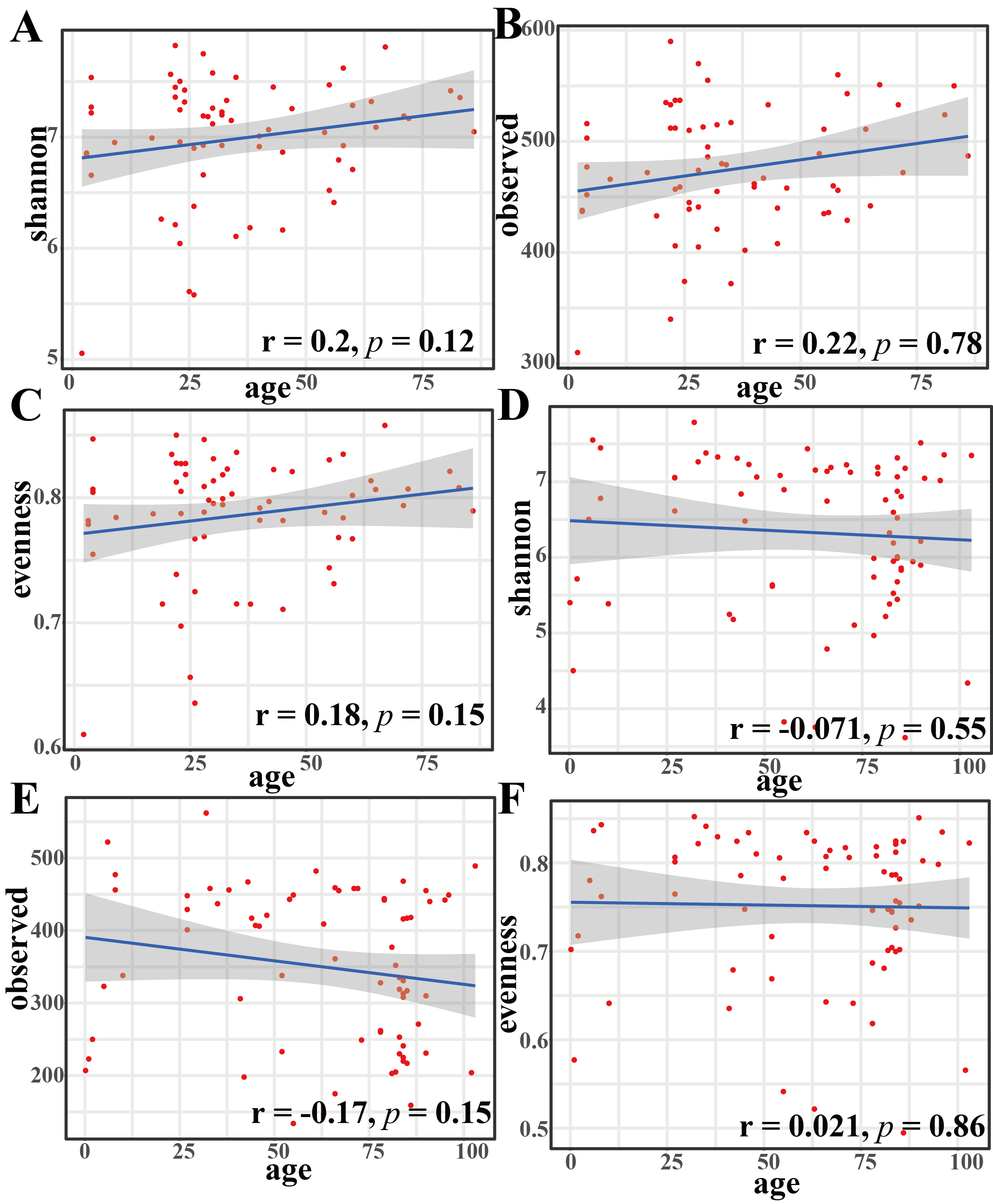
Supplementary Fig. 3 Correlation analysis of gut microbiota Alpha diversity with age in high and low-altitude population**

(A-C) The correlation between Shannon, observed and evenness index and age in Study3 HT population was indicated, respectively.

(D-F) The correlation between Shannon, observed and evenness index and age in Study4 LH population was indicated, respectively.

**
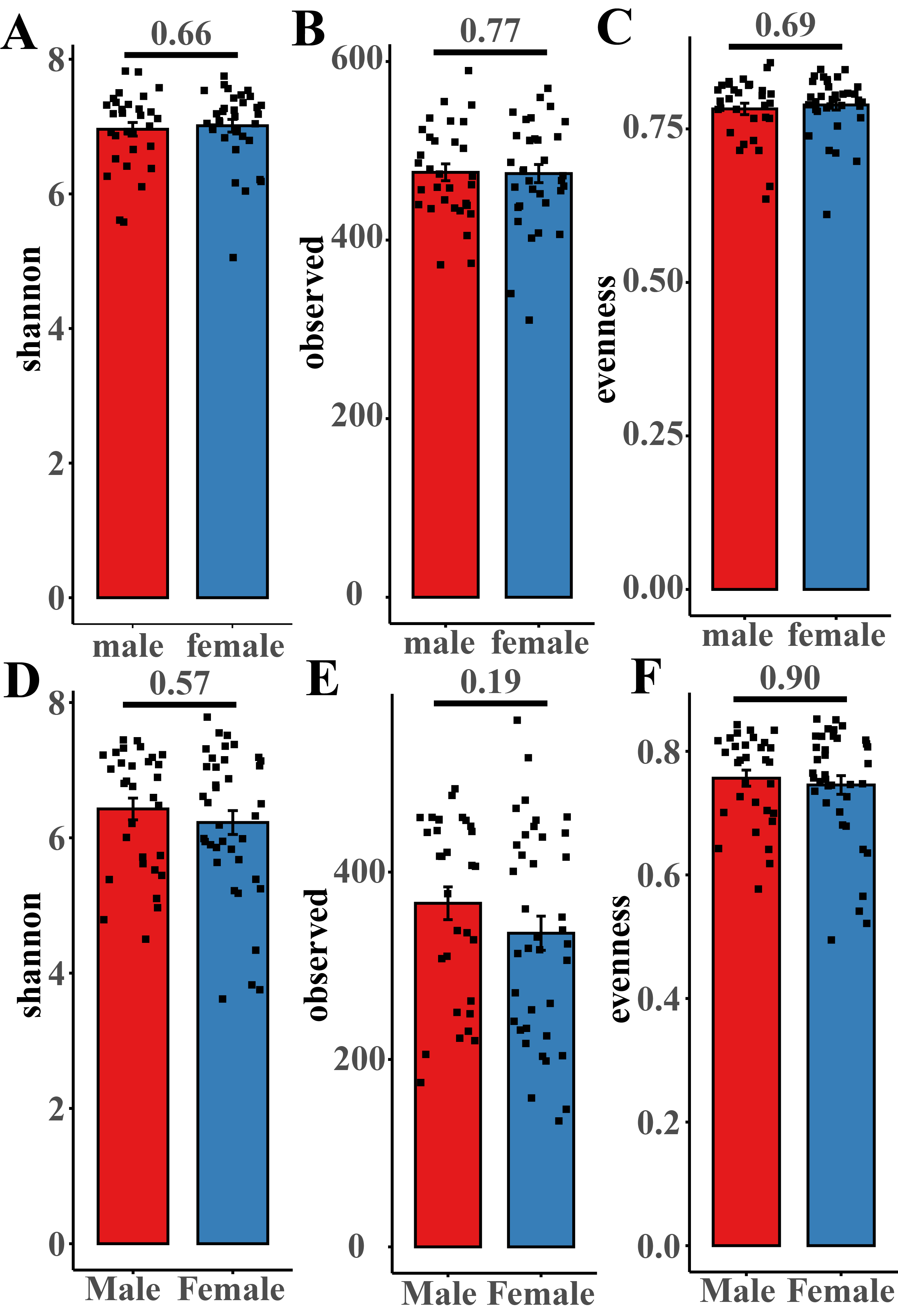
Supplementary Fig. 4 The difference in gut microbiota alpha diversity between high and low-altitude populations of different sex**

**(A-C)** In the study3, the differences between Shannon, observed, and evenness index among HT populations of different sex, respectively. **(D-F)** In the study4, the differences between Shannon, observed, and evenness index among LH populations of different sex, respectively.


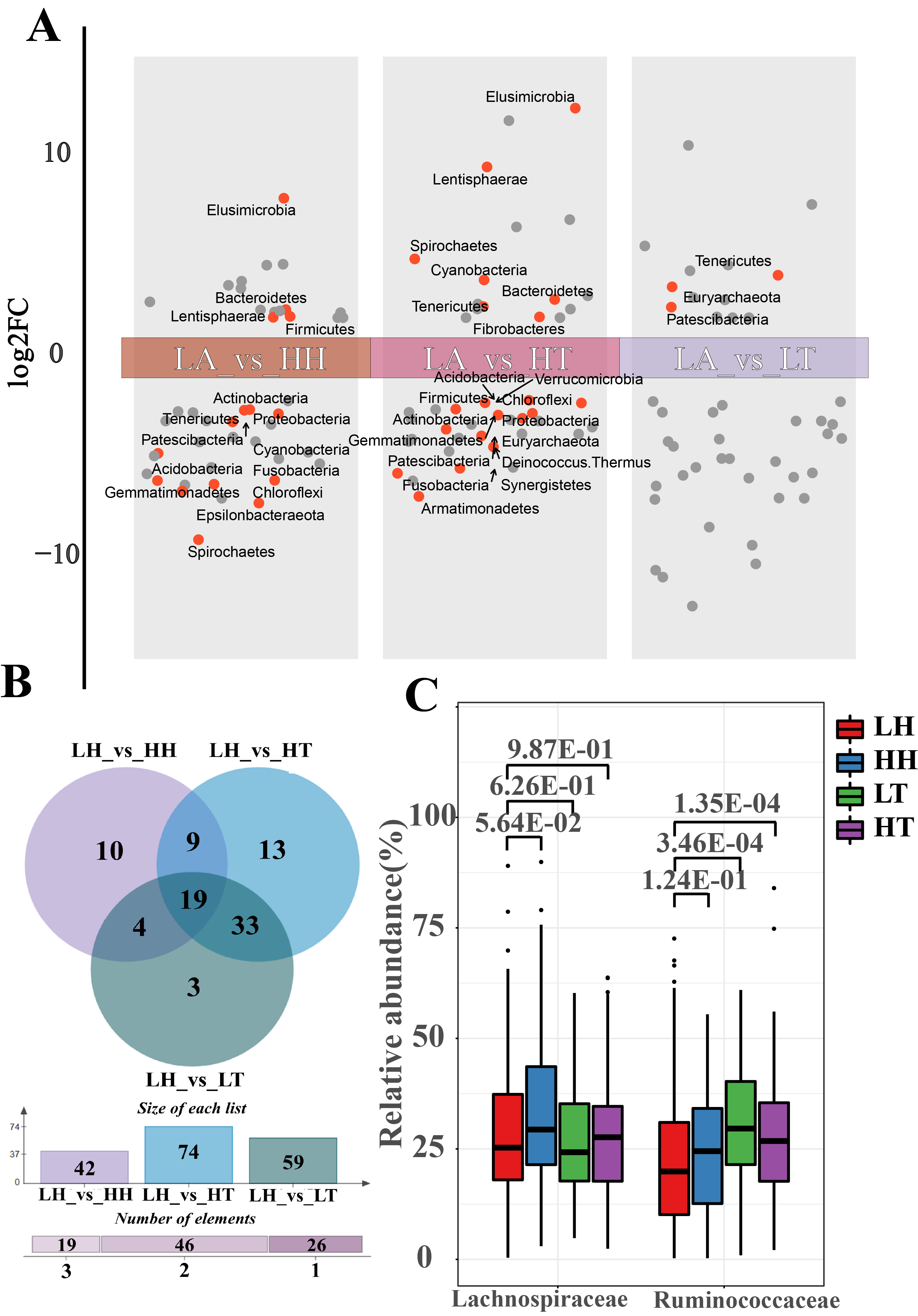
**Supplementary Fig. 5 Differences in gut microbiota between high- and low-altitude people.**

**(A)** LH_vs_HH, LH_vs_HT, and LH_vs_LT phylum level difference volcano map. A red dot indicates a significant difference (FDR-corrected *p* value < 0.05), while a gray dot indicates no significant difference. The ordinate represents Log2 (Flod Change), where a value greater than 0 indicates enrichment in HH, HT, or LT, and less than 0 indicates enrichment in LH. **(B)** LH_vs_HH, LH_vs_HT, and LH_vs_LT genera level difference Venn map. The middle bar chart shows the significant differences between the genera LH_vs_HH, LH_vs_HT, and LH_vs_LT, respectively. The bottom bar chart shows the number of different genera shared within 3,2 or 1 circle, respectively. FDR-corrected *p*-value was from a Wilcoxon Rank sum test. The picture is drawn through the E Venn (http://www.ehbio.com/test/venn/#/). (C) Relative abundance of Lachnospiraceae and Ruminococcaceae in high and low-altitude populations. The p-value was from a Wilcoxon Rank sum test. FDR-corrected *p* value < 0.05 is considered a significant difference.


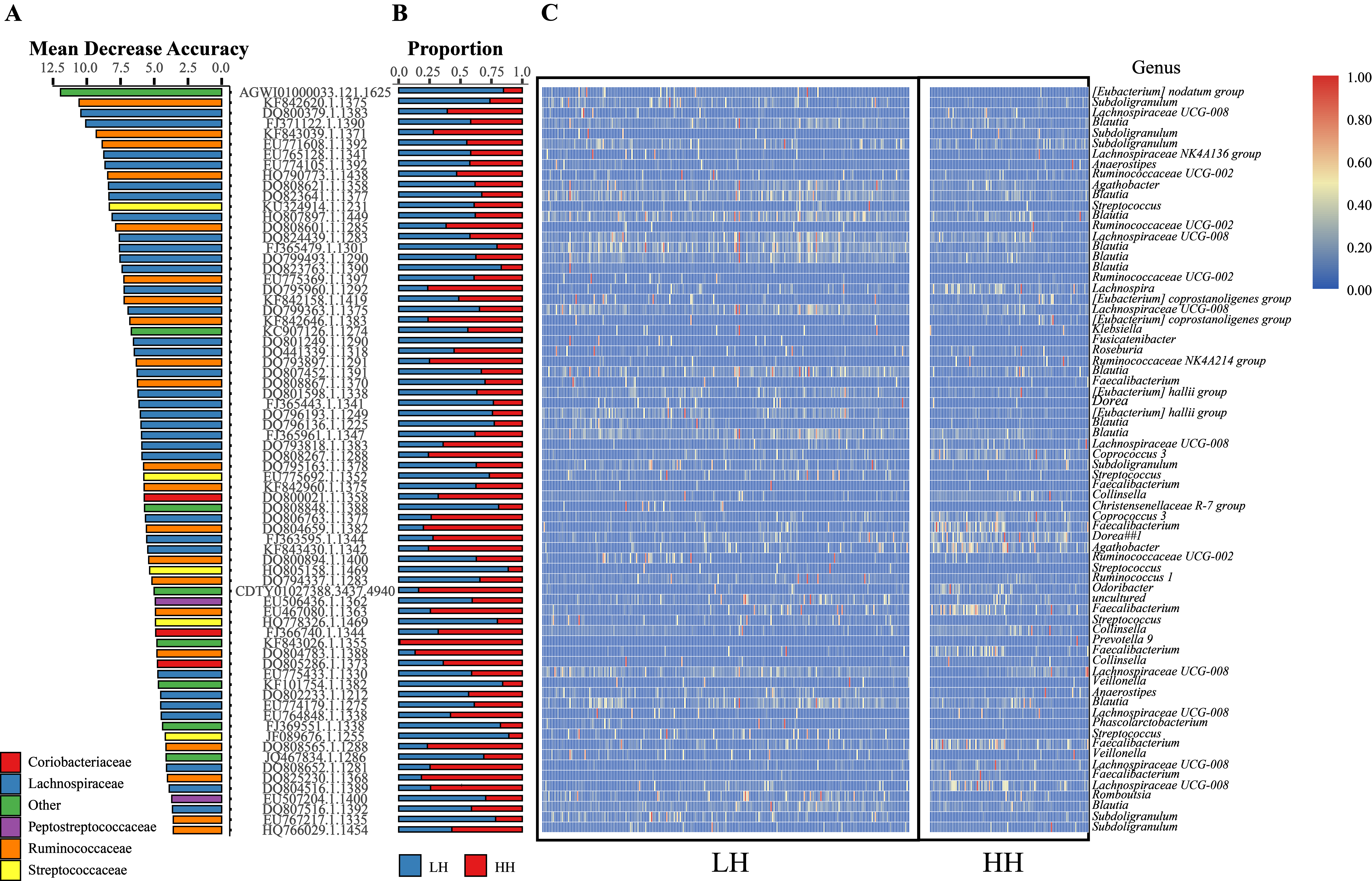


**Supplementary Fig. 6. Gut microbiota biomarkers of LH and HH.**

**(A)** Seventy-two gut microbiota biomarkers are ranked in descending order of importance to the accuracy of the bacterial model. Color represents the family of OTUs classified. **(B)** The relative proportion of the gut microbiota biomarkers. Relative proportion = average relative abundance / (LH average relative abundance +HH average relative abundance). **(C)** Heatmap analysis of the abundance of gut microbiota biomarkers (LH, n=243; HH, n=105)


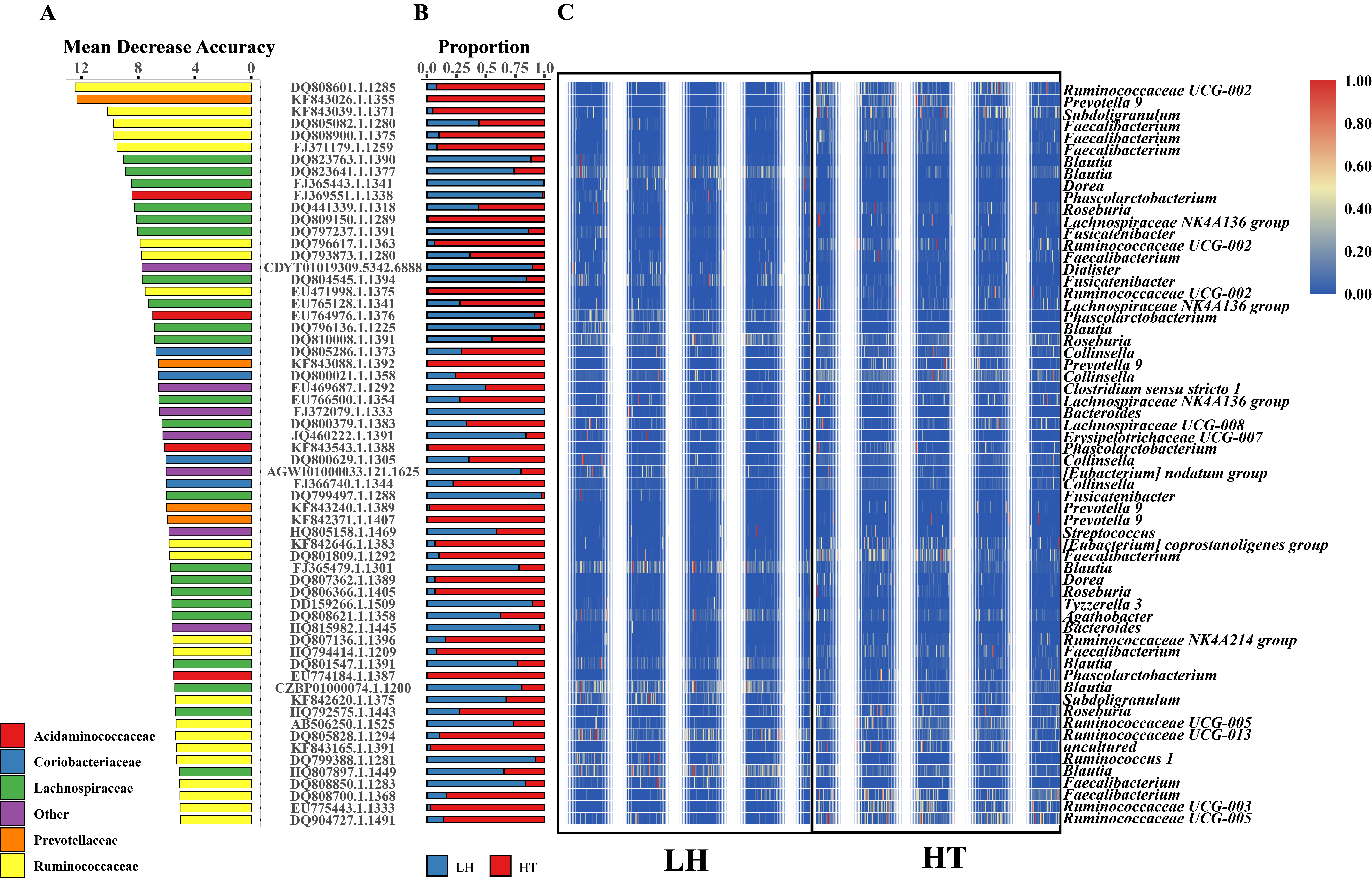


**Supplementary Fig. 7. Gut microbiota biomarkers of LH and HT.**

**(A)** Sixty-two gut microbiota biomarkers are ranked in descending order of importance to the accuracy of the bacterial model. Color represents the family of OTUs classified. **(B)** The relative proportion of the gut microbiota biomarkers. Relative proportion = average relative abundance / (LH average relative abundance +HT average relative abundance). **(C)** Heatmap analysis of the abundance of gut microbiota biomarkers (LH, n=243; HT, n=240)


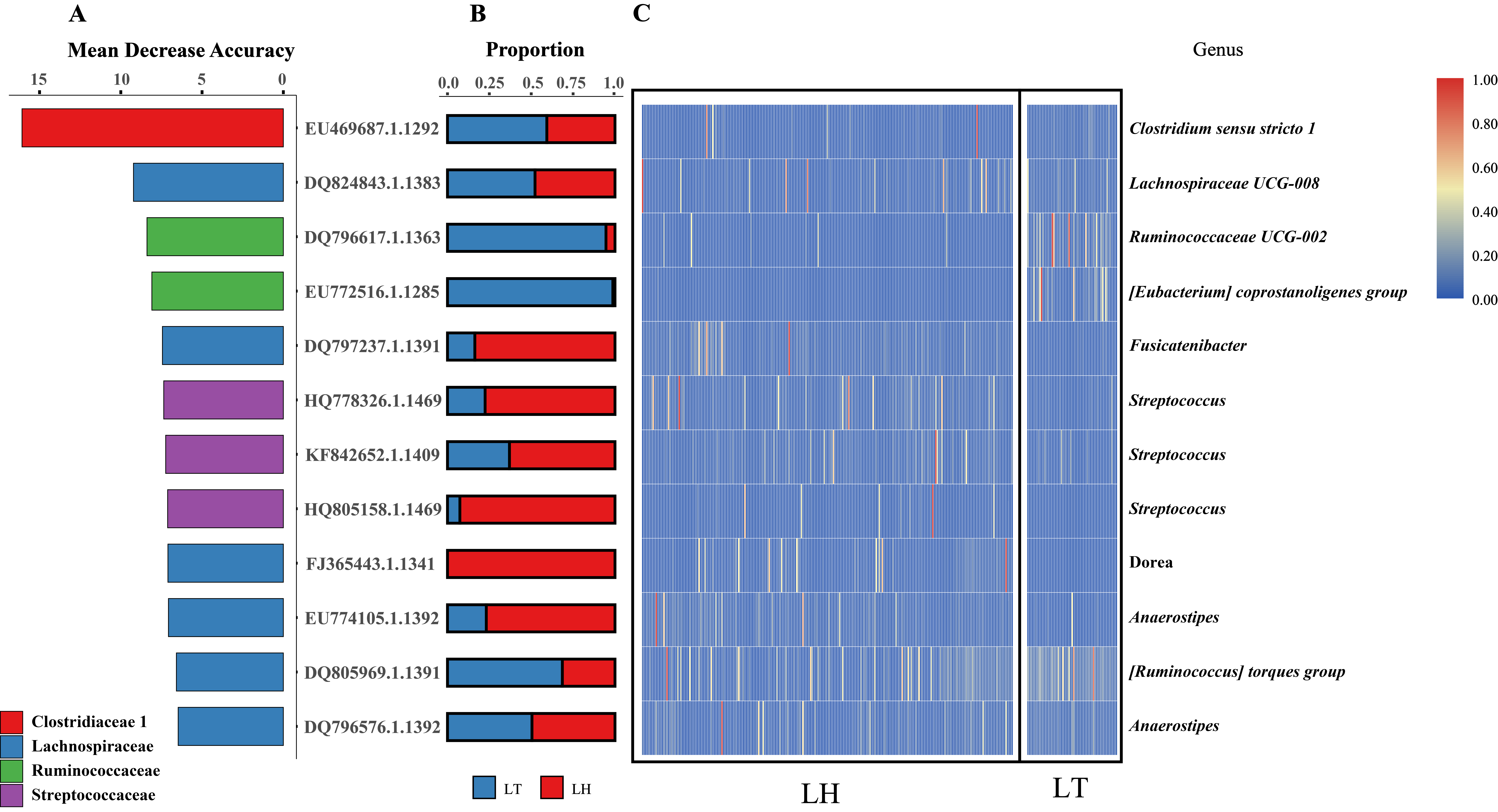


**Supplementary Fig. 8 Gut microbiota biomarkers of LH and LT.**

**(A)** Twelve gut microbiota biomarkers are ranked in descending order of importance to the accuracy of the bacterial model. Color represents the family of OTUs classified. **(B)** The relative proportion of the gut microbiota biomarkers. Relative proportion = average relative abundance / (LH average relative abundance +LT average relative abundance). **(C)** Heatmap analysis of the abundance of gut microbiota biomarkers (LH, n=243; LT, n=59)


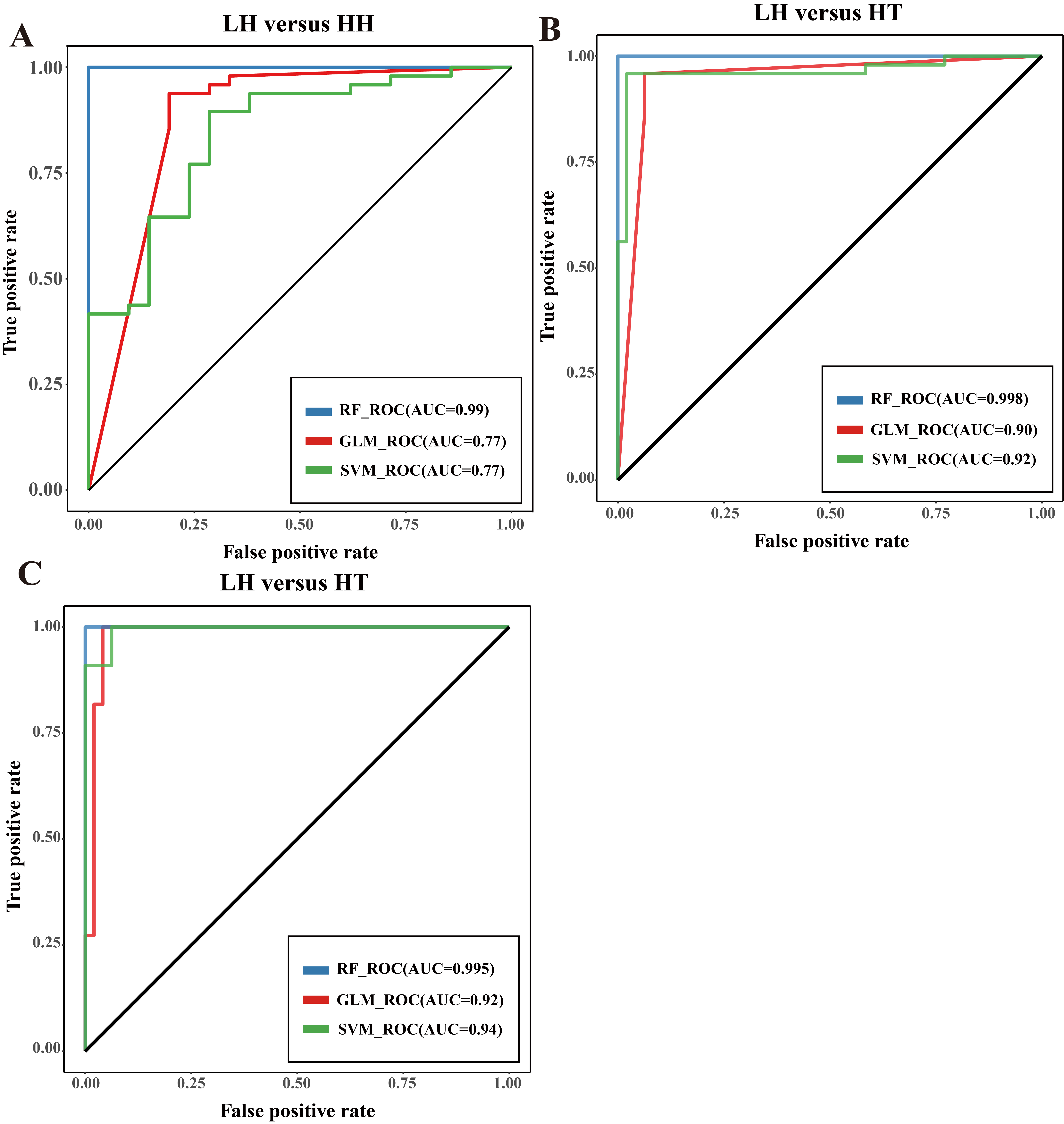
**Supplementary Fig. 9 ROC curves of RF, GLM, and SVM models constructed by gut microbiota of high and low altitude population**

**(A-C)** ROC curves of LH_vs_HH (A), LH_vs_HT (B), and LH_vs_LT (C). 20% of the total samples were randomly selected as the test set, and the remaining samples were used as the training set, repeated ten times. Blue, red, and green represent the ROC curves of the model constructed by RF, GLM, and SVM. Only the three models' optimal ROC curves and average AUC values are shown here.

**Supplementary Fig 10 RF classification model performance at different classification levels.**


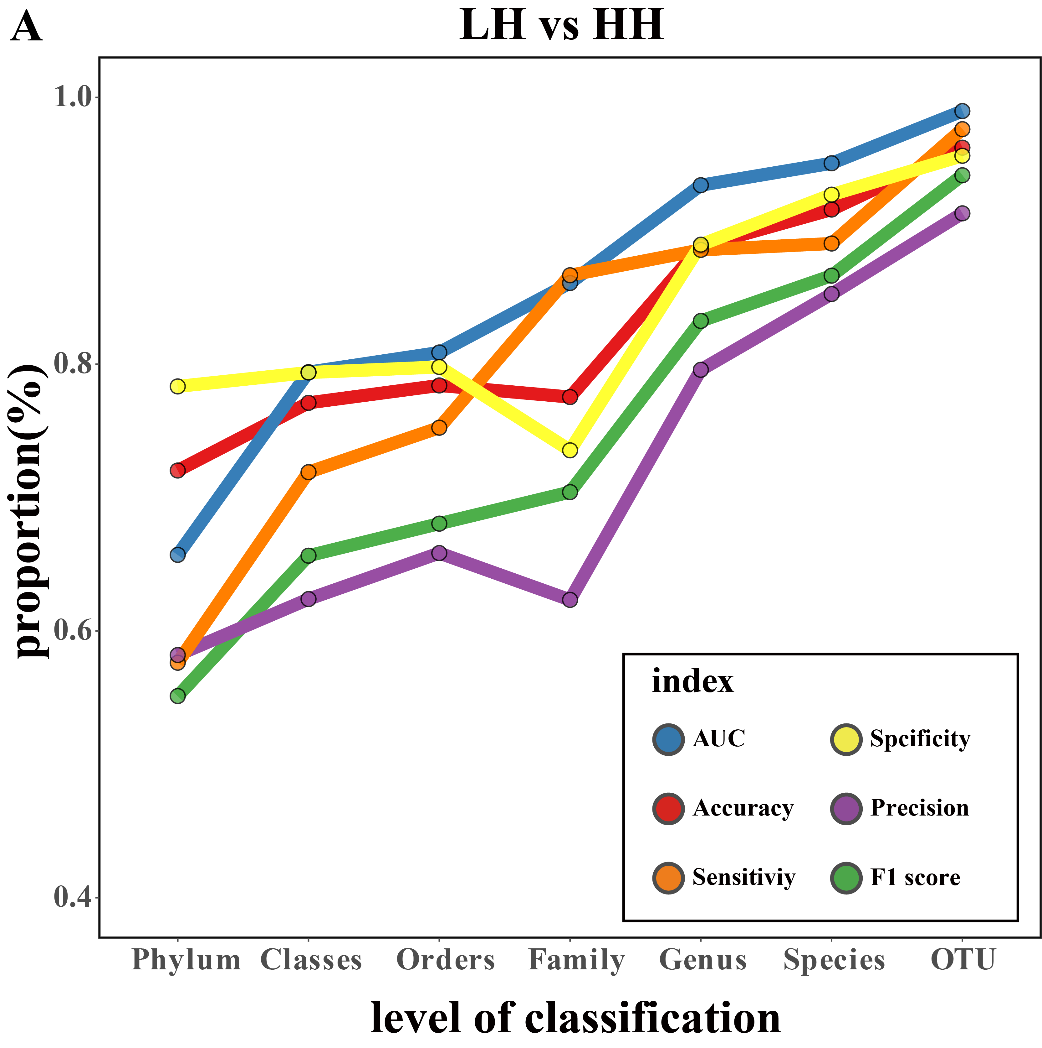

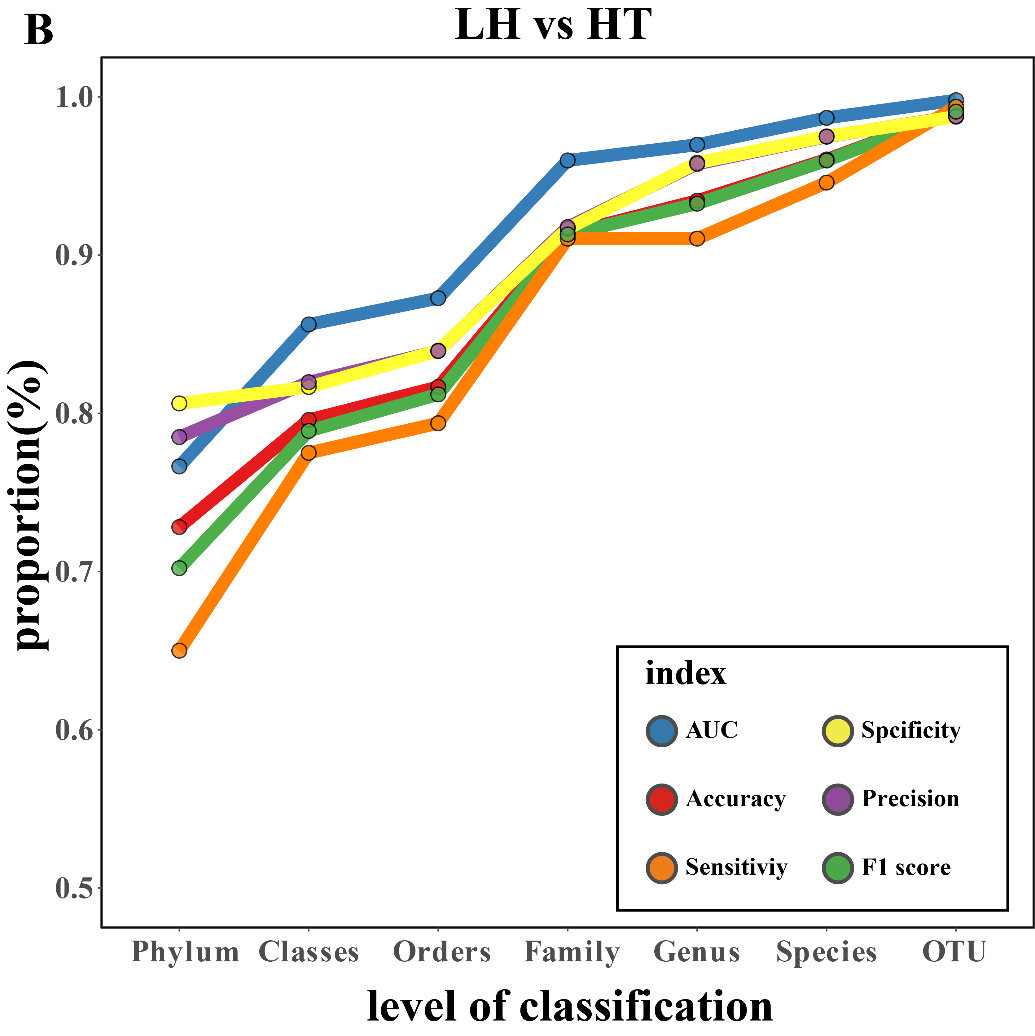

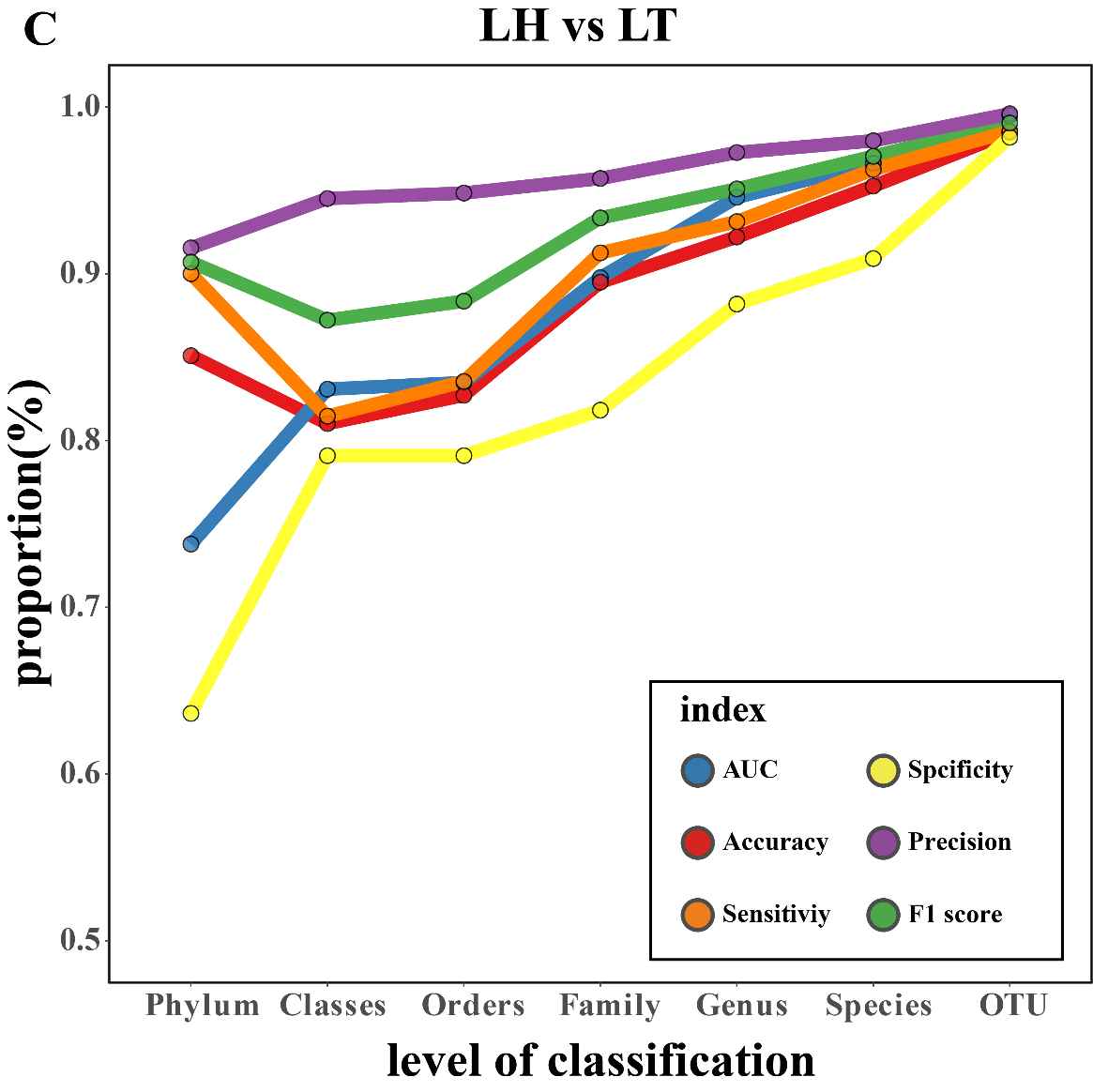


**(A, B, C)** LH_vs_HH (A), LH_vs_HT (B), and LH_vs_LT (C) RF models were constructed at the level of phylum, class, order, family, genus, species, and OTU. 20% of the total samples were randomly selected as the test set, and the remaining samples were used as the training set, repeated ten times. The final evaluation was based on the mean AUC value, Accuracy, Sensitivity, Specificity, Precision, and F1 score of each level marker training model. Blue, red, orange, yellow, purple, and green represent AUC, Accuracy, Sensitivity, Specificity, Precision, and F1 Score, respectively.


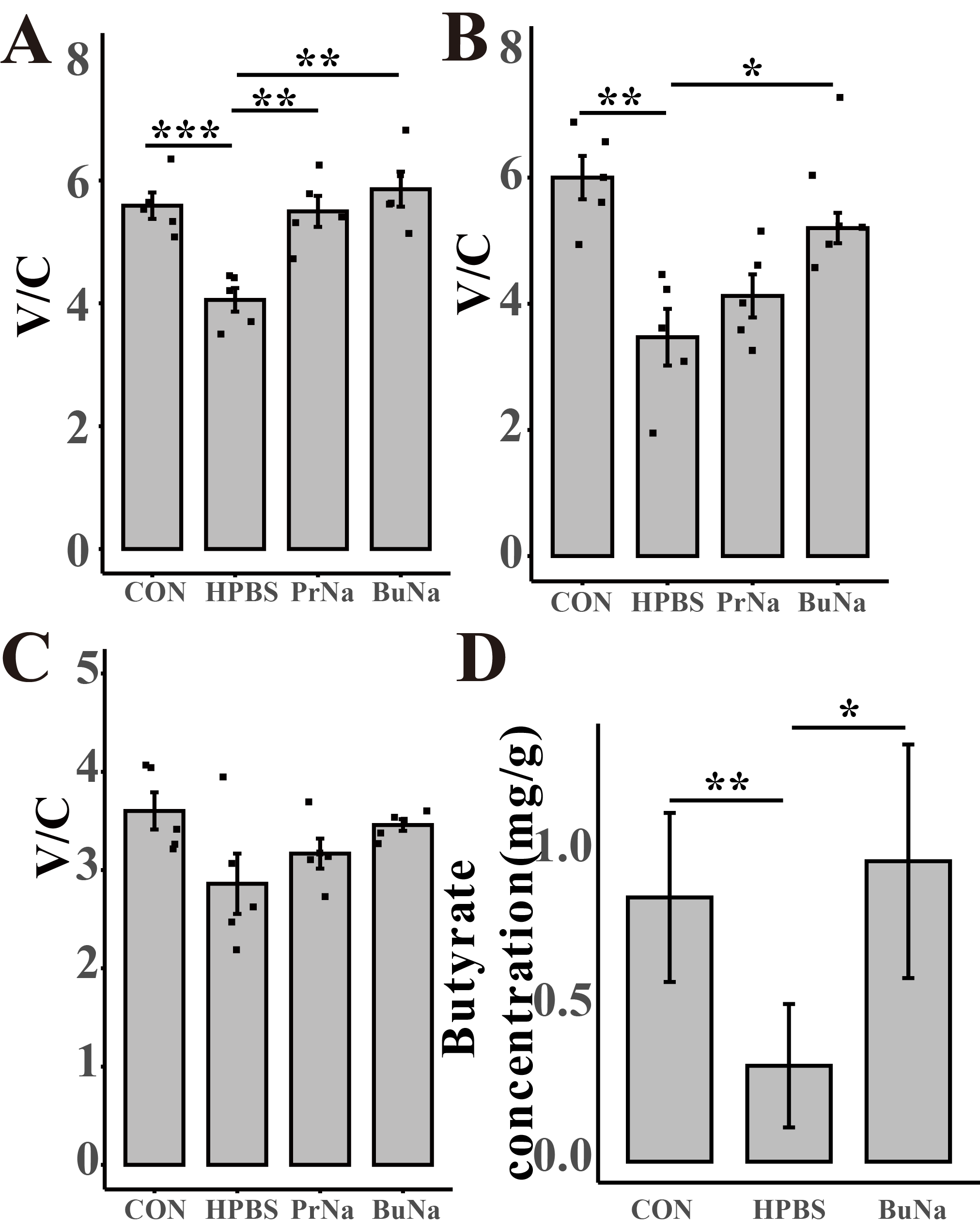


**Supplementary Fig 11 Intestinal villus length to crypt ratio and fecal butyrate concentration in high and low altitude rats.**

**(A-C)** The villus (V) length ratio to crypt (C) depth in the duodenum, jejunum, and ileum, respectively. **(D)** Fecal butyrate concentration. Con: control group; HPBS: high-altitude PBS group; BuNa: high-altitude butyrate group; PrNa: high-altitude propionate group. The results are expressed as Mean±SE (n=5), and different letters between columns indicate significant differences. “*” stands for significant difference. *: p < 0.05. **: p < 0.01. ***: p < 0.001. **** p < 0.0001. The p value is calculated by t-test.

**Supplementary Fig. 12 Cell viability was measured by MTT.**


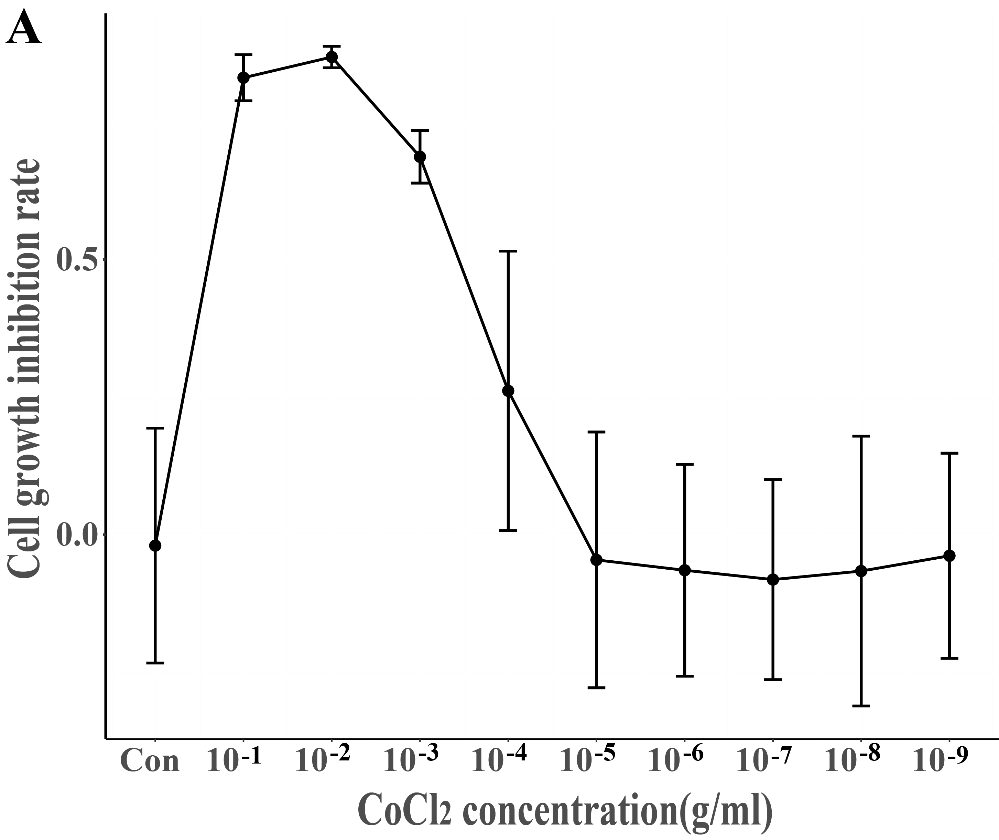

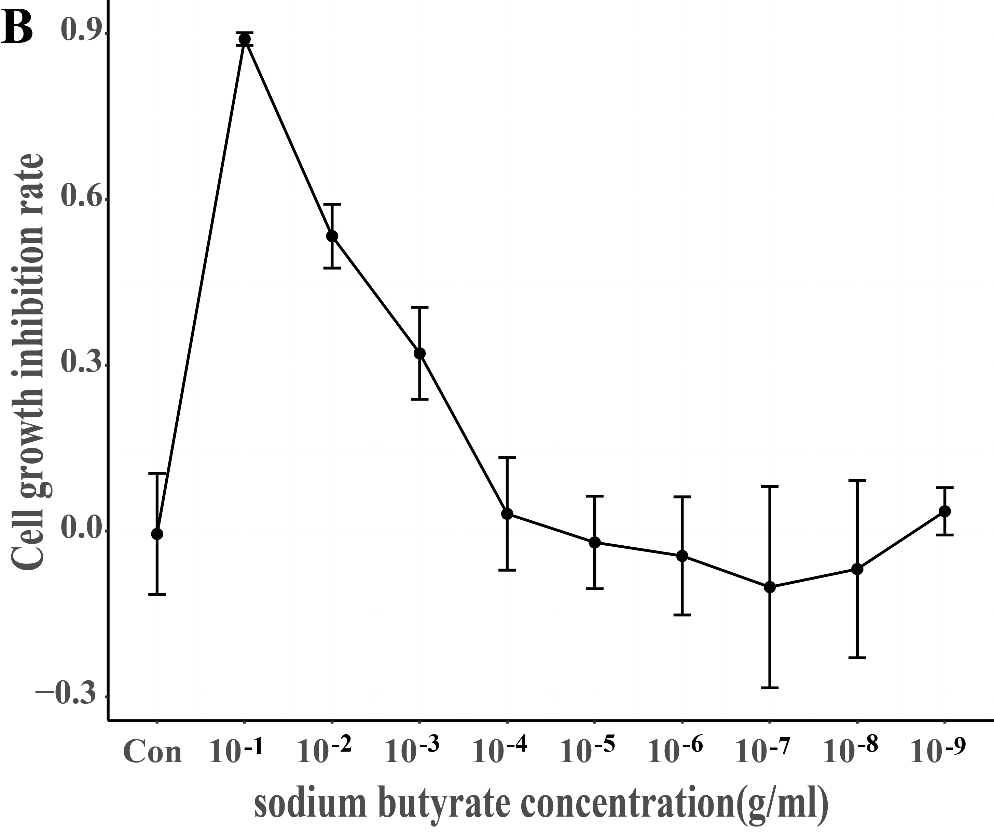


**(A)** Effect of CoCl_2_ treatment on cell viability (n = 6). **(B)** Effect of butyrate treatment on cell viability (n = 6).


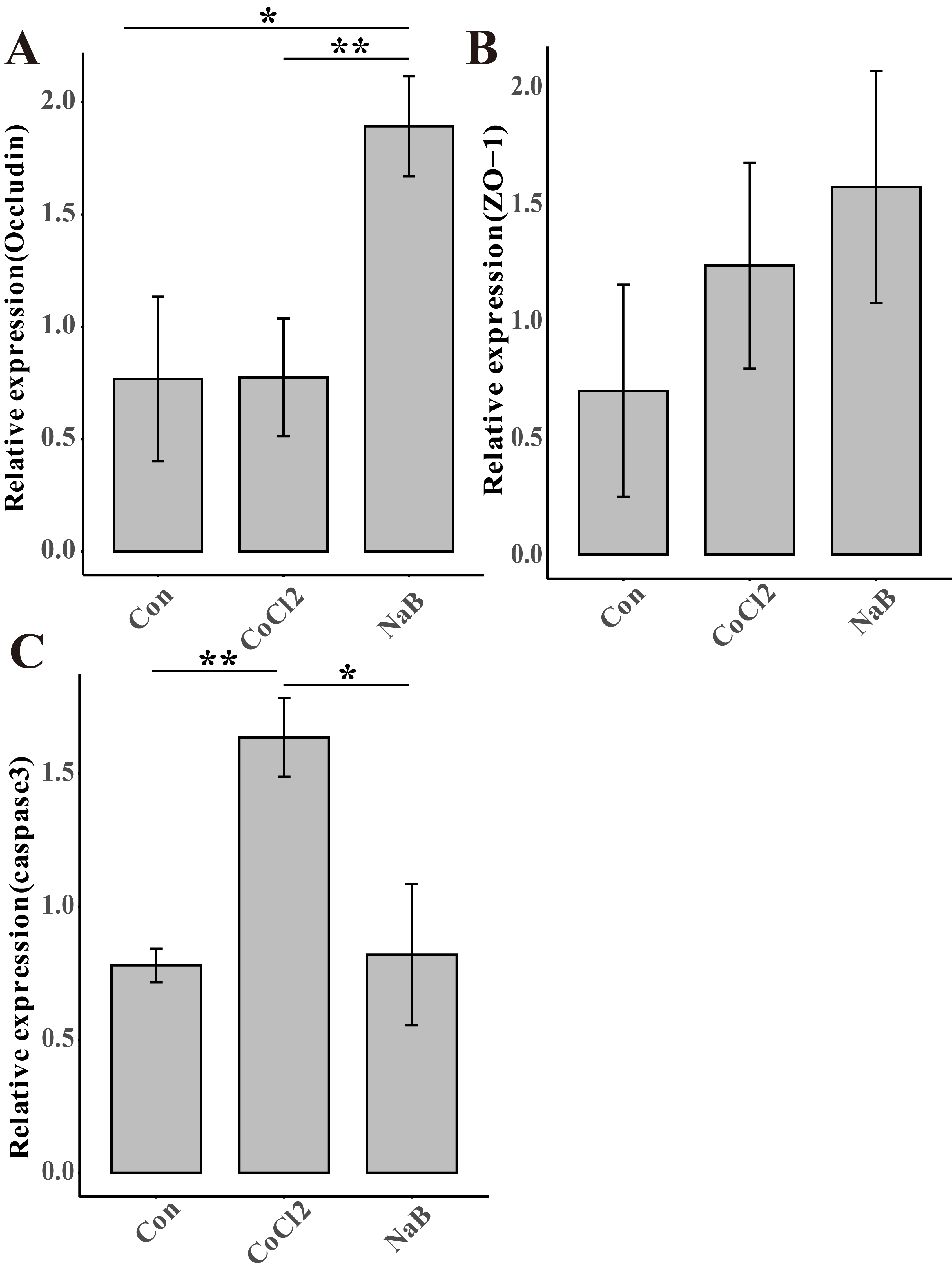
**Supplementary Fig. 13 Changes of gene transcription levels in NCM460 cells treated with butyrate**

**(A)** mRNA expression of *occluding* (Con: n=3; CoCl_2_: n=3; NaB: n=3). **(B)** mRNA expression of *ZO-1* (Con: n=3; CoCl_2_: n=3; NaB: n=3). **(C)** mRNA expression of *caspase3* (Con: n=3; CoCl_2_: n=3; NaB: n=3). “*” stands for significant difference. *: p < 0.05. **: p < 0.01. ***: p < 0.001. **** p < 0.0001. The p value is calculated by t-test.


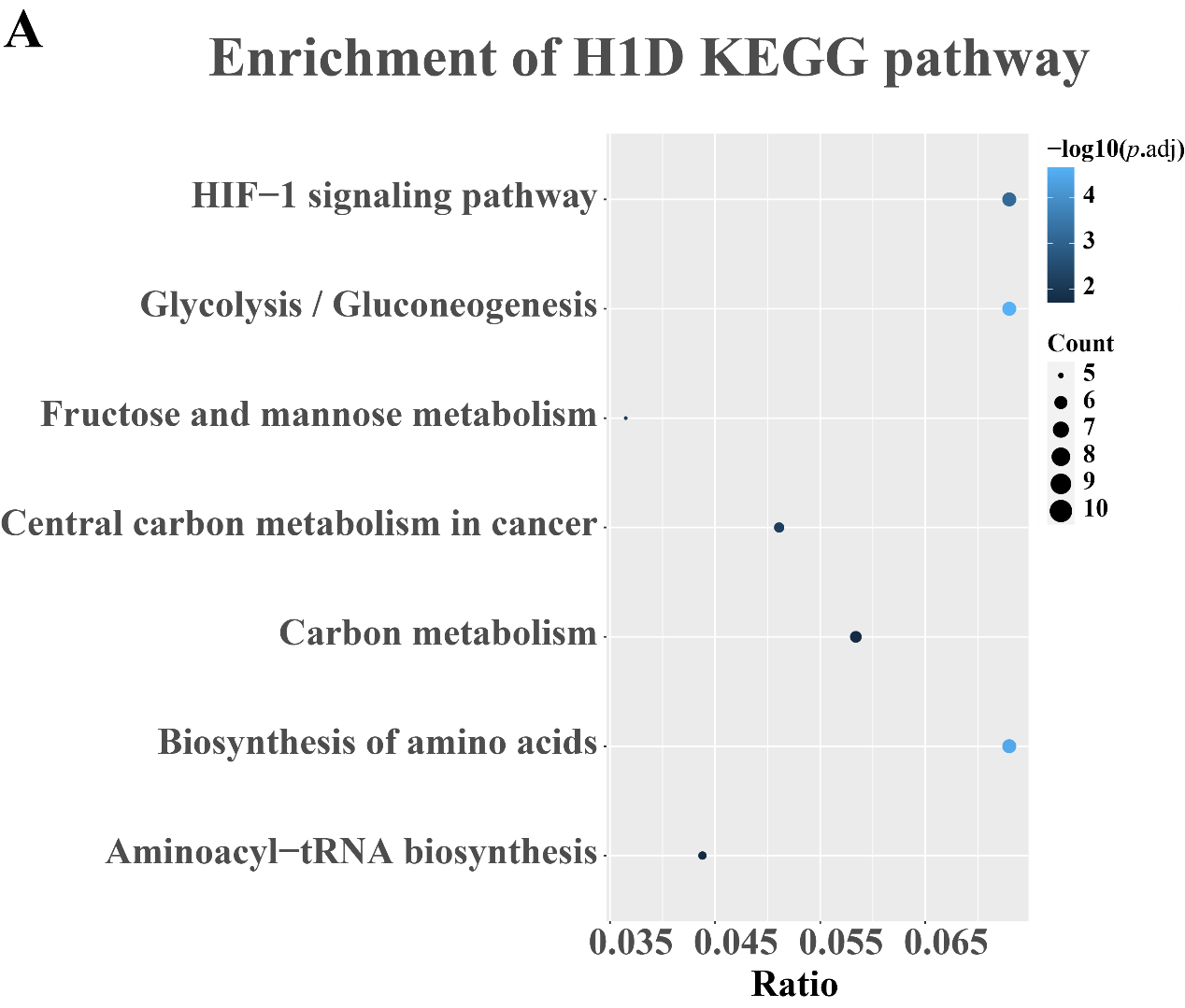


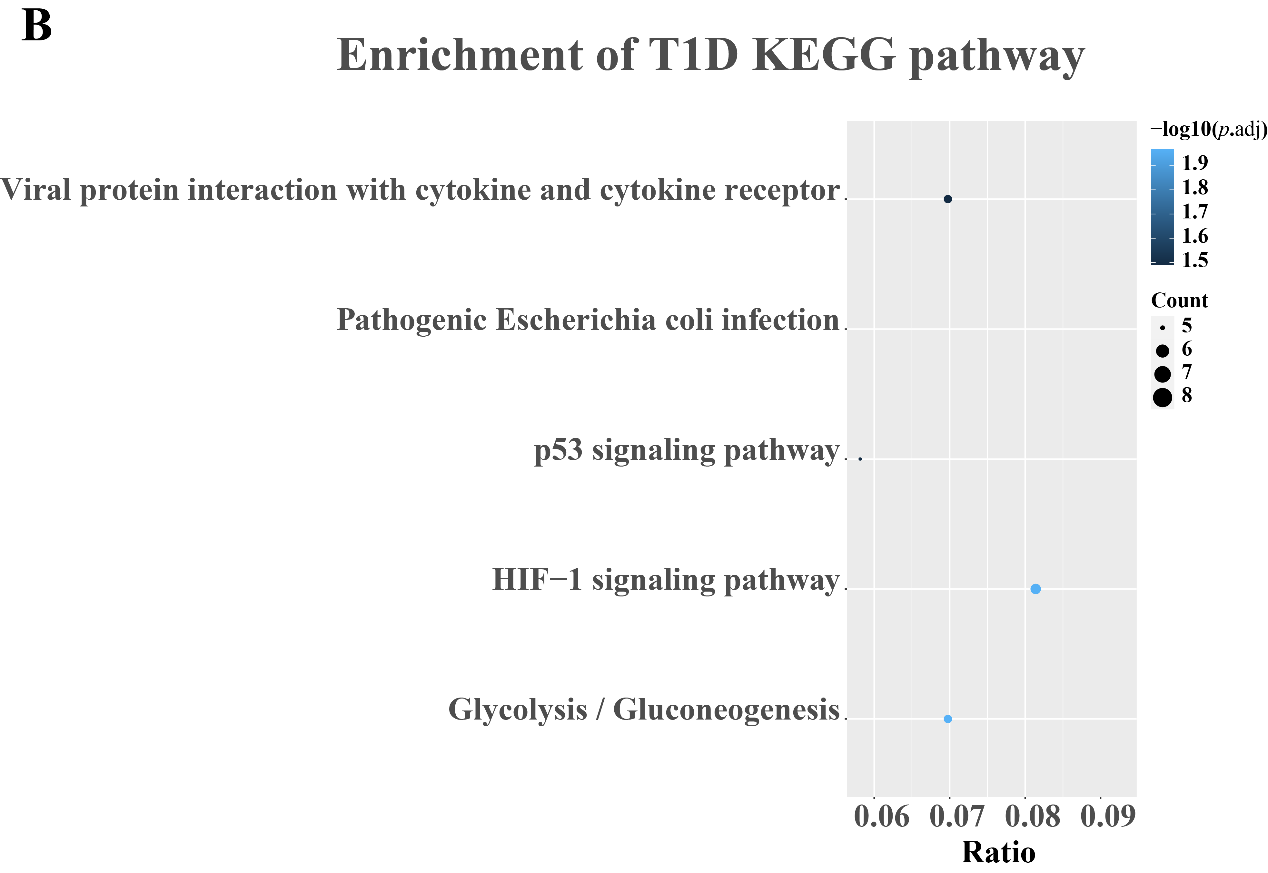


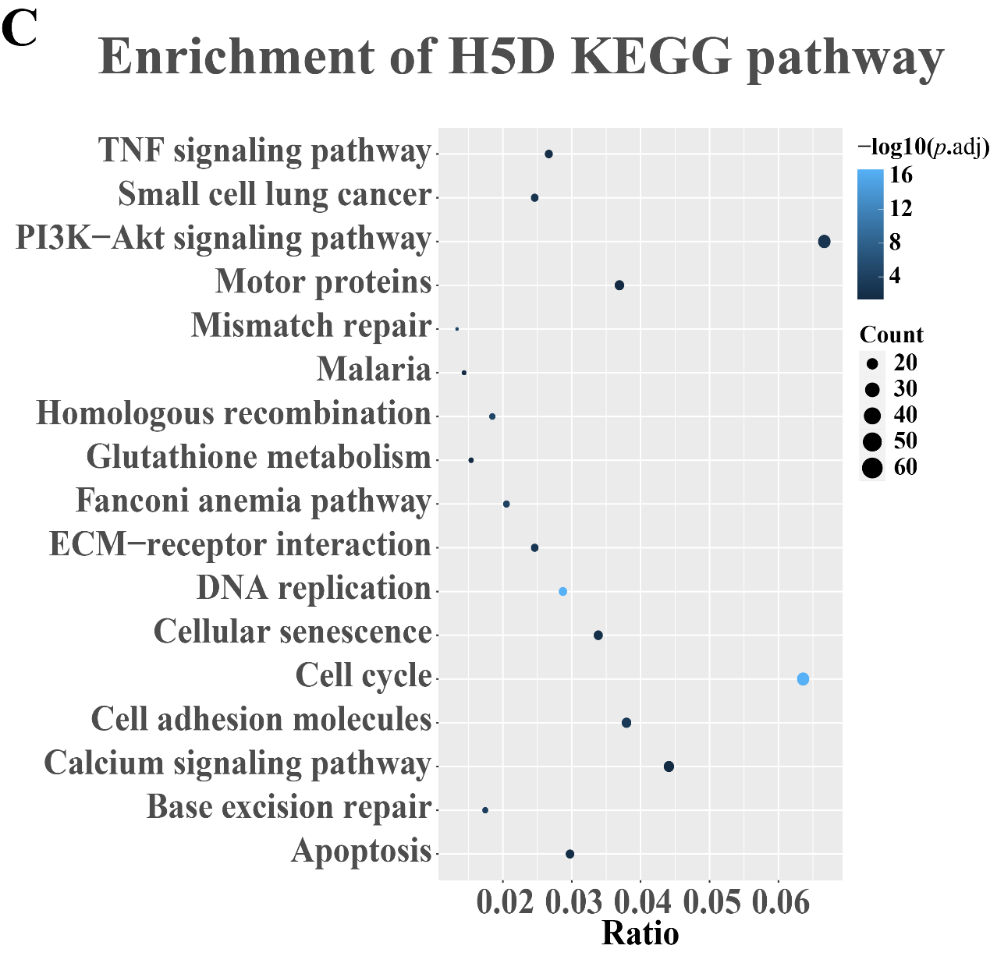


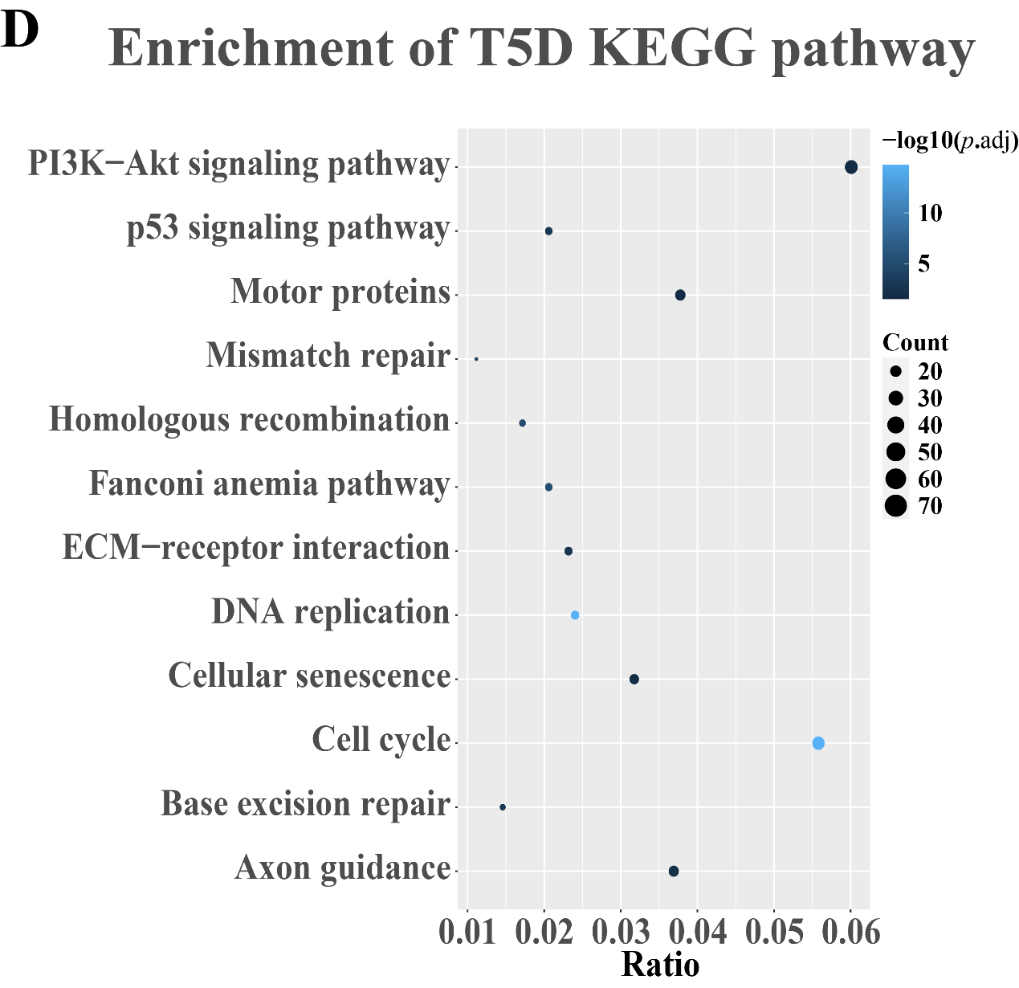


**Supplementary Fig. 14 Enrichment of KEGG pathway at different times of hypoxia in** **Tibetan and Han** **HUVEC cells.**

**(A)** One day of hypoxia in Han HUVEC cells (n = 5). **(B)** One day of hypoxia in Tibetan HUVEC cells (n = 5). **(C)** Five days of hypoxia in Han HUVEC cells (n = 5). **(D)** Five days of hypoxia in Tibetan HUVEC cells (n = 5).


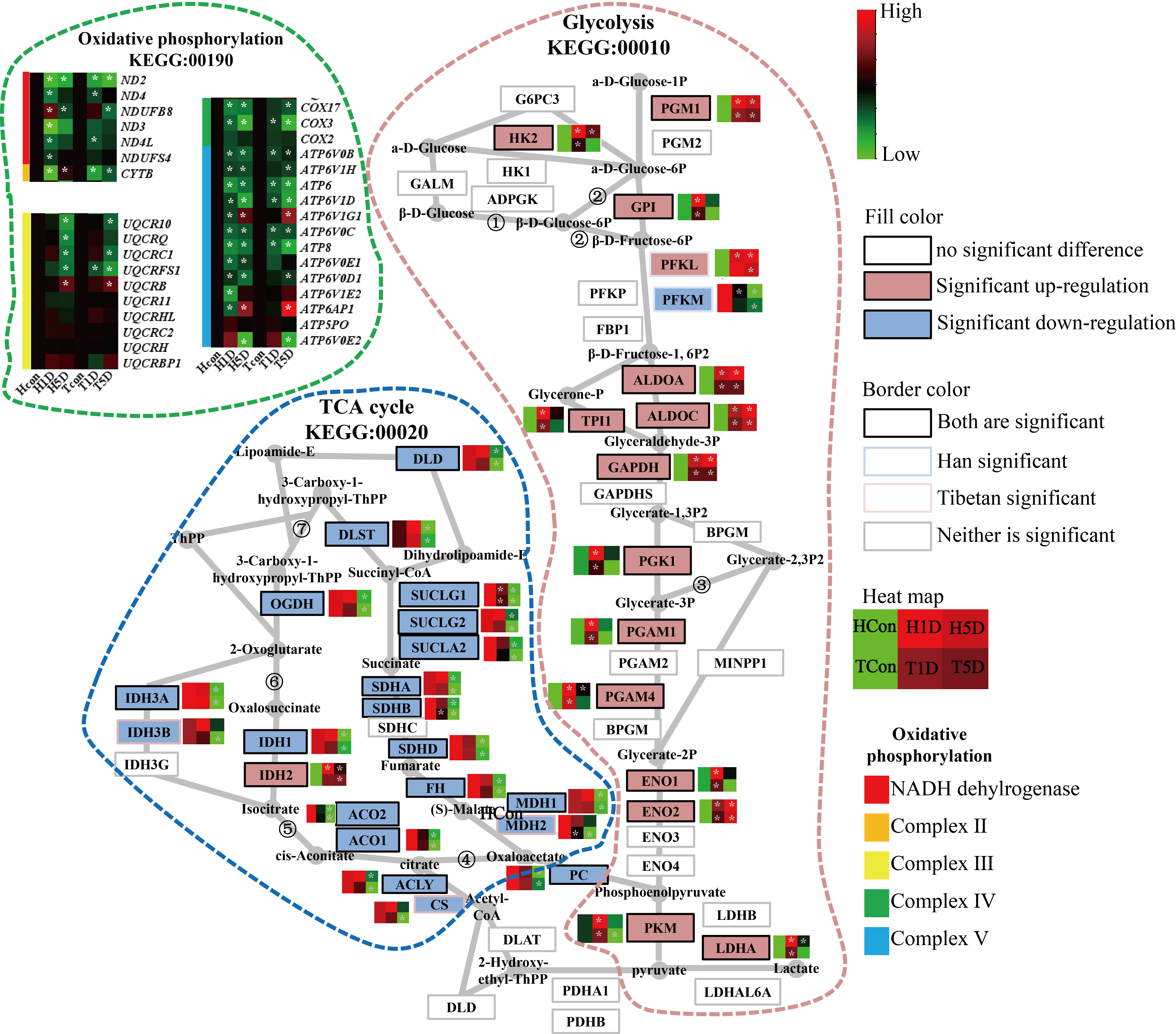


**Supplementary Fig. 15** Transcription analysis of Glycolysis, TCA cycle, and Oxidative phosphorylation pathway in Tibetan Han HUVEC cells after hypoxia treatment. The squares show the changes in genes, and Glycolysis shows the changes in genes after a day of hypoxia. The TCA cycle indicates the change of cells after five days of hypoxia. Heat maps show changes in gene expression. ①: HK1, HK2 and ADPGK; ②: GPI; ③: PGK1; ④: CS and ACLY; ⑤: ACO1 and ACO2; ⑥: IDH1 and IDH2; ⑦: OGDH

**
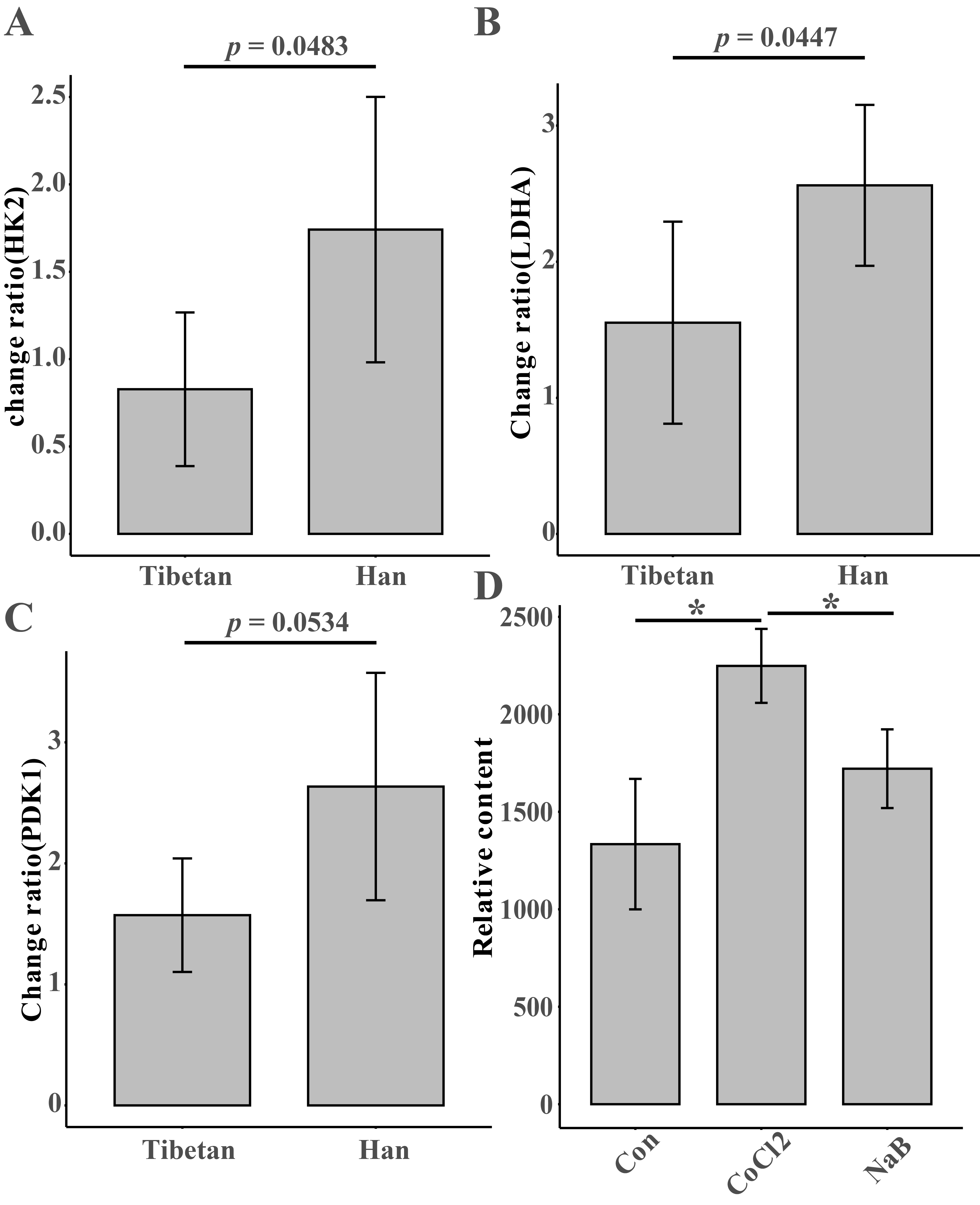
Supplementary Fig. 16 Changes of key genes of glycolysis pathway in** **Tibetan and Han HUVEC cells after 1 day of hypoxia.**

**(A)** *HK2* gene changes (Tibetan, n = 5; Han, n = 5), *p* values were obtained from a two-sided t-test. **(B)** *LDHA* gene changes (Tibetan, n = 5; Han, n = 5), *p* values were obtained from a two-sided t-test. **(C)** *PDK1* gene changes (Tibetan, n = 5; Han, n = 5), *p* values were obtained from a two-sided t-test. **(D)** Intracellular lactate concentration (Con: n=3; CoCl2: n=3; NaB: n=3).

“*” stands for significant difference. *: p < 0.05. **: p < 0.01. ***: p < 0.001. **** p < 0.0001. The p value is calculated by t-test.

**
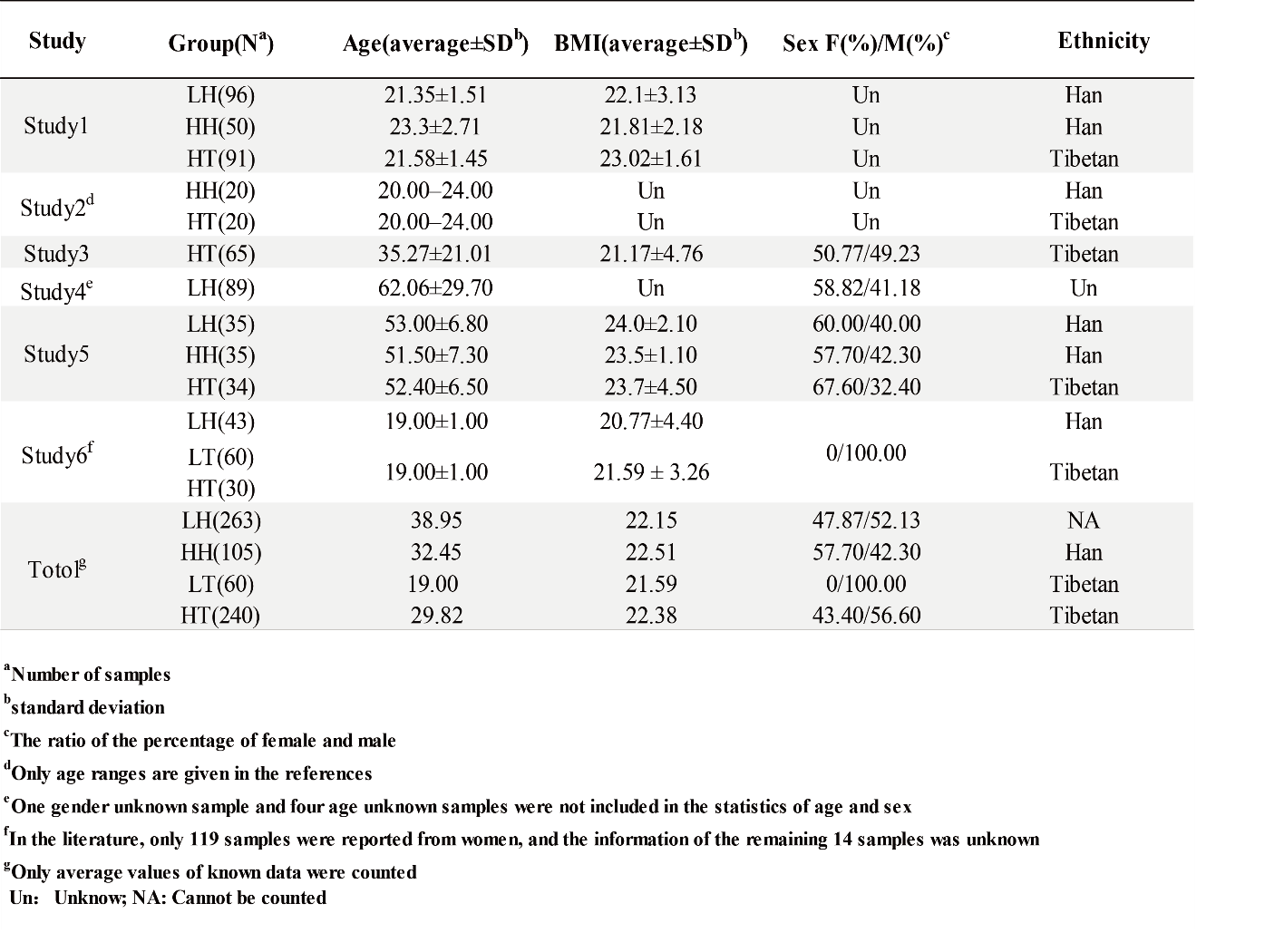
Supplementary Table 1. Characteristics of data sets included in this study.**

**Supplementary Table 2. The difference between different "Group" on different PCoA Axis before ConQuR processing**


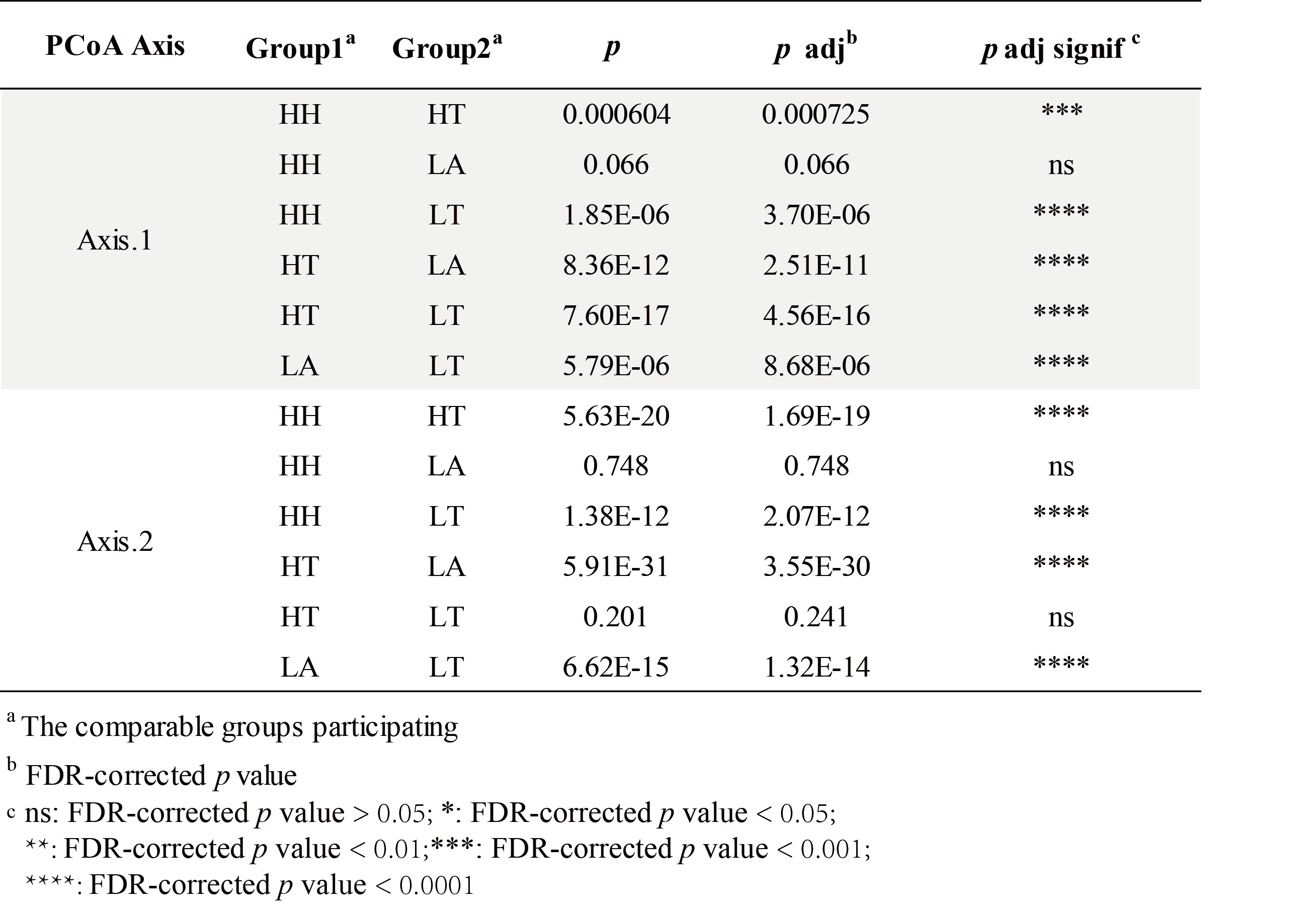


**Supplementary Table 3. The difference between different "Study" on different PCoA Axis before ConQuR processing**

**
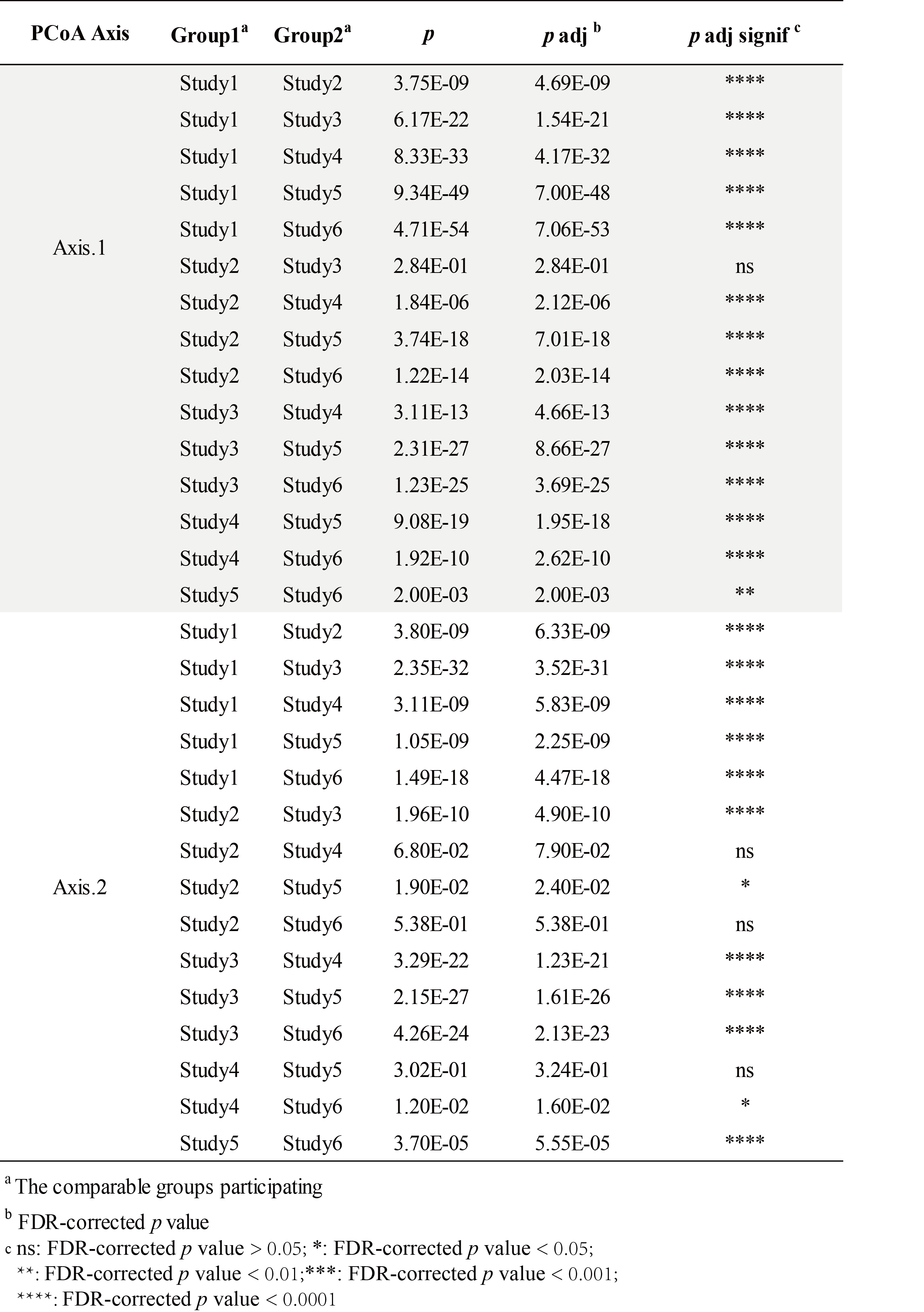
**

**Supplementary Table 4. The difference between different "Group" on different PCoA Axis after ConQuR processing**

**
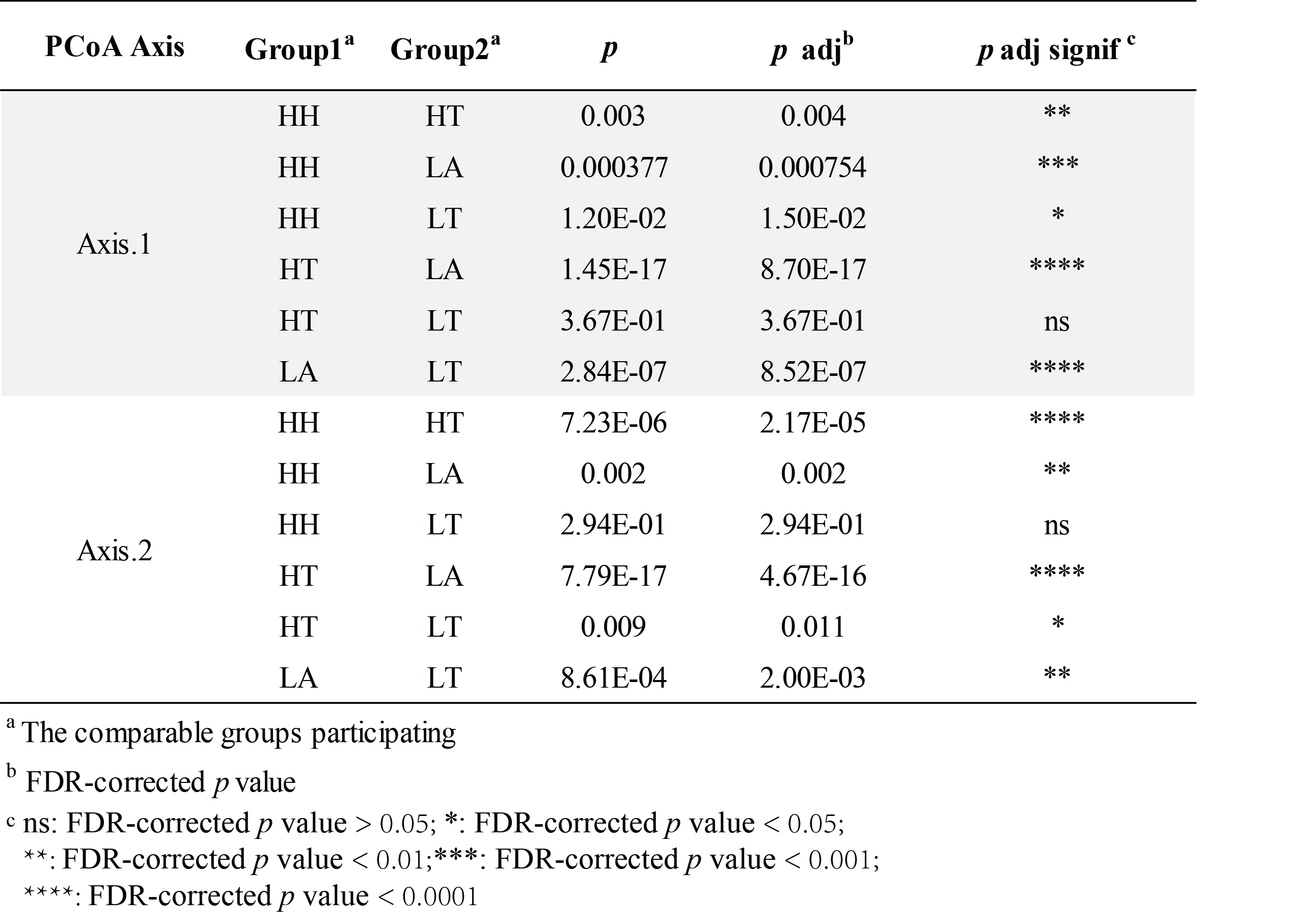
**

**Supplementary Table 5. The difference between different "Study" on different PCoA Axis after ConQuR processing**


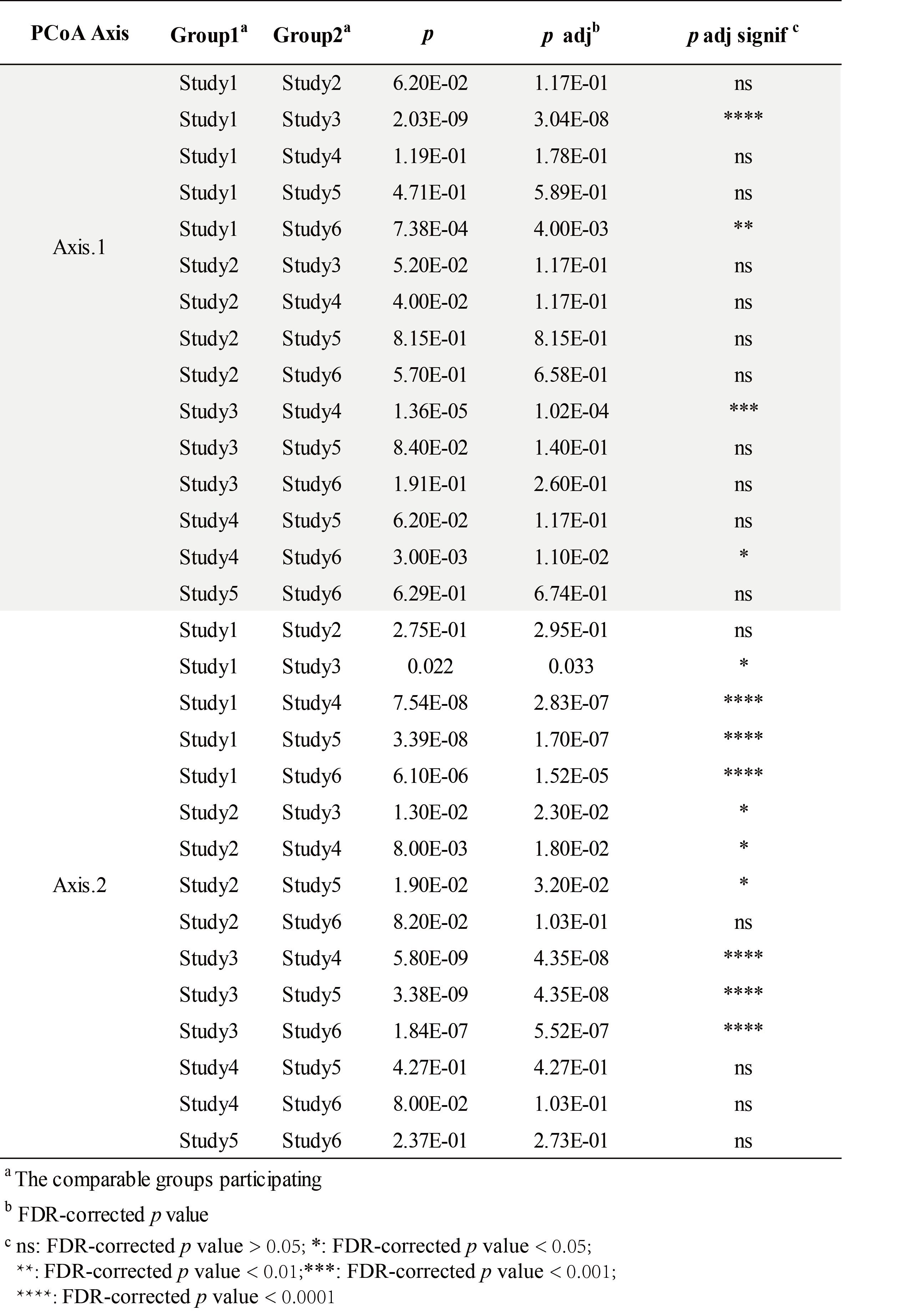


**Supplementary Table 6. Primer sequence**


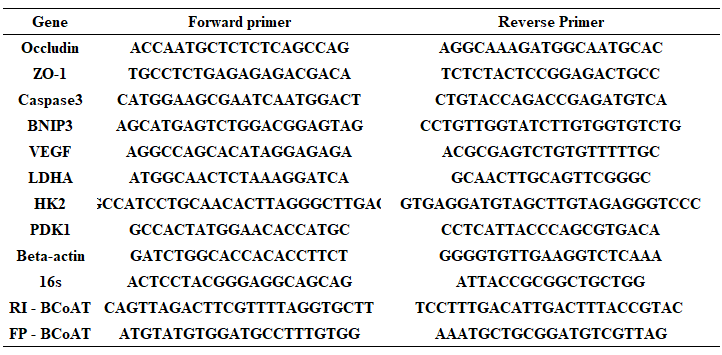

Supplement: Supplemental Material [file KGMI_A_2350151_SM6862.zip › Revised Supplementary Material.docx]
